# Supplementary material for: Secure privacy-preserving record linkage system from re-identification attack
Source: PLoS One. 2025 Jan 9;20(1):e0314486. doi: 10.1371/journal.pone.0314486 (PMC11717198; doi:10.1371/journal.pone.0314486)
Supplement: Attachment — Submitted filename: [PLOS ONE]Reply Letter.pdf [file pone.0314486.s003.pdf]

Reply Letter

Manuscript ID: **PONE-D-23-43609**

Title: **Secure privacy-preserving record linkage system from re-identification attack**

Authors: Sejong Lee, Yushin Kim, Yongseok Kwon, Sunghyun Cho*

Dear Associate Editor and Reviewers,

Most of all, we would like to thank the associate editor and the reviewers for spending their valuable time and effort reviewing our paper. We have carefully read all of the reviewers' comments and have revised our manuscript according to reviewers' insightful comments and suggestions. Please find below our detailed replies to each of the comments.

Once again, we appreciate your kind and careful suggestions.

Sincerely,

Sunghyun Cho, Prof./Ph.D.

Dept. of Computer Science and Engineering.
Hanyang University ERICA
55 Hanyangdaehak-ro, Sangrok-gu, Ansan, Gyeonggi-do, Korea.

Tel: +82-31-400-5670 / Fax: +82-31-436-8152
Email: chopro@hanyang.ac.kr

Authors' Response to Editor and Reviewers' Comments (Manuscript ID: PONE-D-23-43609)

[Response to Reviewer 1 Comments]

[Comment 1-1]
The title of this paper, you must modify. Short form is not allowed in title.

(Response 1-1)
We appreciate the reviewers' valuable comments.

In response to Comment 1-1 regarding the title of our paper, we have revised it to eliminate the short form and ensure clarity and formality.

We hope this modified title meets the expectations and standards of the journal.

The details of the revisions are as follows:

(Revision 1-1)

Before	After
**[Title, p.1]**<br>Secure privacy-preserving record linkage (PPRL) system from re-identification attack	**[Title, p.1]**<br>Secure privacy-preserving record linkage system from re-identification attack

[Comment 1-2]
Improve your abstract section.

(Response 1-2)
We appreciate the reviewers' valuable comments.

In response to comments 1-2 regarding the abstract section, we have revised the abstract to be concise and clearer.

The details of the revisions are as follows:

(Revision 1-2)

Before	After
[Abstract, p.1]<br>Recent studies have revealed that privacy-preserving record linkage (PPRL) technology is vulnerable to graph-based re-identification attacks. This poses a severe security issue for PPRL, which links similar records across multiple data sets while preserving privacy. Graph-based re-identification attack techniques infer source records by contrasting	***[Abstract, p.1]***<br>Privacy-preserving record linkage (PPRL) technology, crucial for linking records across datasets while maintaining privacy, is susceptible to graph-based re-identification attacks. These attacks compromise privacy and pose significant risks, such as identity theft and financial fraud. This study introduces a novel zero-relationship encoding scheme designed

plain text graphs with encoded record graphs, which can neutralize the privacy-preserving technology of PRRL and steal privacy. Leaked privacy can be exploited for crimes, such as identity theft and financial fraud, causing severe harm to people. Therefore, a new PPRL technology that can effectively resist re-identification attacks and securely preserve user privacy is necessary. This study proposes a zero-relationship encoding scheme to prevent record re-identification attacks in PPRL systems. The proposed zero-relationship encoding method minimizes the relationship between the source and encoded records, preventing an attacker from inferring the source record. To verify the privacy-preserving performance of the proposed method, a record re-identification attack simulation was performed using the Titanic and North Carolina Voter Records (NCVR) datasets. Simulation results show that an average of 16 (1.21\%) of the 1317 records in the Titanic data set were re-identified. For the NCVR dataset, all 4401 records resisted the re-identification attack, achieving a re-identification rate of 0\%. Security analysis proves zero-relationship encoding preserves user privacy by defending against graph-based re-identification attacks.	to enhance PPRL systems' resistance to re-identification attacks by minimizing the linkage between source and encoded records. Our method's efficacy was validated through simulations on the Titanic and North Carolina Voter Records (NCVR) datasets, demonstrating a substantial reduction in re-identification rates. Security analysis confirms that our zero-relationship encoding effectively preserves privacy against graph-based re-identification threats, improving PPRL technology's security.

[Comment 1-3]
Which are you used software for simulation. Show the simulation results.

(Response 1-3)
We appreciate the reviewers' valuable comments.

The content within Section 4.1, "Simulation Setup," has been revised in response to the comments concerning the simulation software utilized.

The details of the revisions are as follows:

(Revision 1-3)

Before	After
[Section 4, p.7-8] Results: Evaluation and Discussion This section evaluates the privacy-preserving performance and feasibility of the proposed zero-relationship encoding method by simulating the re-identification attack outlined in [18].	***[Section 4, p.7-8]*** 4. Results: Evaluation and Discussion In this section, we conduct experiments using a graph-based re-identification attack simulator to evaluate the proposed zero-relationship encoding method's privacy preservation performance and validity.

Simulation Setup

This study simulates a scenario in which an attacker attempts to re-identify anonymized records using a plain data set. All simulations were performed on Ubuntu 20.04.1 with an Intel(R) Xeon(R) W-3245 3.20 GHz CPU and 256 GB of memory. Two datasets were used in the security durability evaluation simulation against re-identification attacks: the Titanic[1] and North Carolina Voter Registration (NCVR) databases[2]. In the Titanic dataset, we used attributes such as the first and last names of 1,317 passengers for encoding. The NCVR dataset, which was created in June 2014, was used in the simulation as both encoding and plain data for re-identification. This dataset includes information on 224,073 voters from North Carolina and comprises 19 attribute fields. However, the NCVR dataset contains numerous null values. Using these null values in the anonymization process can introduce noise or errors into the results. Therefore, to ensure an accurate simulation, we used information from 4,401 voters that did not contain any null values. In the simulation using the NCVR dataset, we randomly selected attributes for encoding from a pool of 17, excluding the two attributes with the highest and lowest cardinality values from a total of 19 user attributes.

4.1. Simulation Setup

The performance of the proposed encoding method was assessed through experiments utilizing a re-identification attack simulator provided by [22]. The simulator, implemented in Python 2.7, was executed on a PC with a 64-bit Intel(R) Xeon(R) W-3245 3.20 GHz CPU and 256 GB of memory, running Ubuntu 20.04.1. Access to the utilized simulator is available via the link provided by the authors at `https://dmm.anu.edu.au/pprlattack/`. For the simulations, two types of public datasets were employed: the Titanic dataset [47] and the North Carolina Voter Registration (NCVR) database [48]. The Titanic dataset utilized attributes such as the first and last names of 1,317 passengers. The NCVR dataset (accessed in June 2014) contains information on 224,073 North Carolina voters across 19 attribute fields. Due to numerous null values across fields, which could introduce noise or errors if used without preprocessing, a subset of 4,401 voter records without any null values was employed for accurate simulation. In the simulations based on the NCVR dataset, attributes with the highest and lowest cardinality values were excluded, and a subset of the remaining 17 attributes was randomly selected for use.

[Section 4.2, p.8]

Figure 3. Re-identification rates in the Titanic dataset: Comparing encoding methods and matching algorithms.

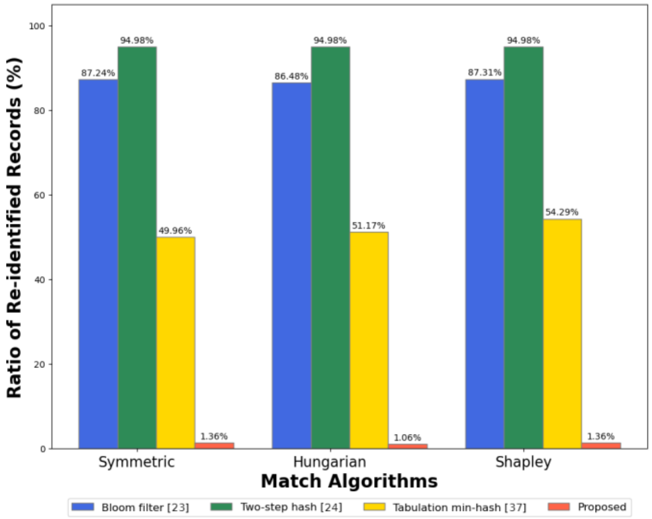

Encoding method	Match Algorithms		
	Symmetric	Hungarian	Shapley
Bloom filter [23]	87.24 % (1149)	86.48 % (1139)	87.31 % (1150)
Two-step hash [24]	94.98 % (1251)	94.98 % (1251)	94.98 % (1251)
Tabulation min-hash [37]	49.96 % (658)	51.17 % (674)	54.29 % (715)
Proposed scheme	1.36 % (18)	1.06 % (14)	1.36 % (18)

Figure 4. Re-identification rates in the NCVR dataset: Comparing encoding methods and matching algorithms.

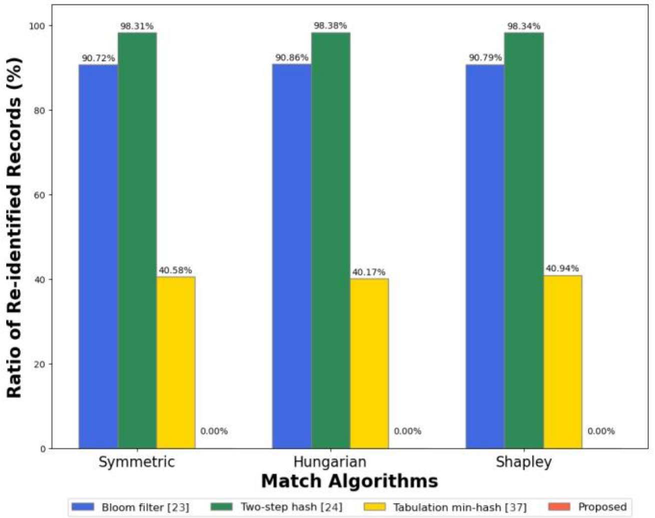

Encoding method	Match Algorithms		
	Symmetric	Hungarian	Shapley
Bloom filter [23]	90.72 % (3993)	90.86 % (3999)	90.79 % (3996)
Two-step hash [24]	98.31 % (4327)	98.38 % (4330)	98.34 % (4328)
Tabulation min-hash [37]	40.58 % (1786)	40.17 % (1768)	40.94 % (1802)
Proposed scheme	0 % (0)	0 % (0)	0 % (0)

[Comment 1-4]

What is the novelty of your work? Include the comparison table with published work.

(Response 1-4)

We appreciate the reviewers' valuable comments.

In response to Comment 1-4 concerning the novelty of our work and the comparative analysis with previously published works, we have incorporated a comprehensive table in the section 2. This table presents a qualitative comparison between our proposed method and existing techniques detailed in relevant literature.

The added comparison table highlights the key features, methodologies, and limitation of our proposed technique alongside those of established methods. We have endeavored to clearly illustrate the innovative aspects of our approach, emphasizing its strengths and improvements over existing solutions. This addition is intended to provide a clearer understanding of our work's novelty and the tangible benefits it offers within the field.

The details of the revisions are as follows:

(Revision 1-4)

Before	After
[-]	***[Section 2, p.5]***

Table 1. Related work to improve the security of PPRL

Year	Author	Description	Limitation
2017	Smith D.	• Proposed tabulation min-hash (TMH) to remedy the BF encoding method vulnerabilities. Privacy preservation by avoiding simple mapping of plain-texts encoded into q-grams.<br>• Utilizes a bit array of length and lookup tables to encode records based on min-hash values of the q-gram set.	Maintaining essential bits for similarity in encoding.
2020	Ranbaduge et al.	• The windowing-based XORing (WXOR) method is proposed to mitigate vulnerabilities inherent in the BF encoding approach.<br>• Employs a sliding window to search for adjacent bit patterns within q-grams, subsequently combining these patterns using XOR to create new ones.<br>• The window size can be adjusted, allowing for pattern size alterations and optimizing the encoding process's efficiency and security.	Relationships between source and encoded records.
2020	Ranbaduge et al.	• Proposed a secure two-step hash encoding method. Hashes private record information into a bit matrix. Each column is hashed to generate an integer set representation of a sensitive input value.<br>• Real data simulations demonstrate improved record linkage quality in large databases.	Relationships between source and encoded records.
2021	Nóbrega et al.	• Assumed an unrealistic adversarial model and proposed an auditable blockchain-based PPRL (ABEL).<br>• Highlights existing PPRL research limitations that rely on unrealistic adversarial models.<br>• To minimize information exchange during the record linkage process, the splitting bloom filters (SBF) approach is proposed, which exchanges only parts of the encoded data, reducing security threats in the event of data breaches.	Re-identification risks arise if a user becomes malicious during BF exchange.
2022	Victor C. et al.	• Proposed an autoencoder-based method transforming BFs into numerical vectors, addressing vulnerabilities in BF encoding.<br>• Enhances security and privacy by preventing known BF attacks, offering a robust solution against re-identification risks.<br>• Data owners train autoencoder models for BF encoding/decoding, ensuring comparability via a mapping function.	Autoencoder sensitivity.
2023	Siyu Y. et al.	• Proposed a PPRL using a Siamese neural network (SNN). SNN involves three main steps: combined bloom filter encoding module (CMF), blocking, and record matching using SNN. CMF combines a flat bloom filter (FBF) for strings and a transform bloom filter (TBF) for numerical attributes.<br>• Offers enhanced security via CMF encoding, precise record matching with SNN, adaptability to diverse data types, and robust performance in scenarios with data errors.	Autoencoder sensitivity.
2023	Frederik A. et al.	• Propose a BF with diffusion (BFD) PPRL scheme that approaches PPRL by extending conventional BFs with a linear diffusion layer.<br>• BFD enhances security against known attacks while preserving or improving linkage quality, potentially as a robust alternative to standard BF-based PPRL.	Complexity in balancing cost and fairness.
2024	Ours	• Proposes a zero-relationship encoding method aimed at preventing re-identification attacks in PPRL and safeguarding privacy.<br>• Involves selecting quasi-identifiers based on their significance and applying XOR-folding to their hash values to generate encoded records, disrupting the re-identification process.<br>• Effectively protects privacy by preventing re-identification attacks and offers high efficiency due to low computational complexity in encoding.	Processing of records with a small number of attributes.

[Comment 1-5]

At least you mention Figures in result section like through flowchart etc.

(Response 1-5)

We appreciate the reviewers' valuable comments.

In response to your guidance, we have thoroughly revised the Results section. We have minimized the repetition of information depicted in figures, reducing unnecessary descriptions. Instead, we now focus on succinct summaries highlighting the key findings and insights the graphical data conveys. This approach follows with your suggestion to let the figures speak for themselves while the text emphasizes the overarching conclusions and their significance within the context of our study.

The details of the revisions are as follows:

(Revision 1-5)

Before	After
[Section 4, p.7-8]<br>**Results: Evaluation and Discussion**<br>This section evaluates the privacy-preserving performance and feasibility of the proposed zero-relationship encoding method by simulating the re-identification attack outlined in [18].	***[Section 4, p.9-10]***

4330, and 4328 records, indicating that most of the records were re-identified. The tabulation min-hash showed improved performance with 1786, 1768, and 1802 re-identified records. However, the proposed zero-relationship method demonstrated complete resistance to re-identification, with zero re-identified records for all matching algorithms. These results indicate that the zero-relationship encoding method offers excellent resistance to re-identification attacks by minimizing the risk of re-identification. Simulation results confirmed that the durability of the zero-relationship encoding method against re-identification attacks increased as the number of attributes used in the encoding process increased. We observed a lower re-identification rate in the experimental results of the NCVR dataset, which used three attributes, compared to the Titanic dataset, which used two attributes. Building on this finding, we designed experiments to quantitatively assess the impact of attribute count on performance metrics such as encoding time, feature extraction time, and re-identification attack time. Fig ?? shows the durability of the zero-relationship encoding method against re-identification attacks depending on the number of attributes. We verified that the increase in the number of attributes used in the encoding process increases the time spent on encoding. The time required for feature extraction and re-identification attacks also increased. Particularly when more than 14 attributes were used with encoding, we observed a sharp increase in the time required. This suggests that using numerous attributes for encoding results in the creation of a form without specific meaning, making feature extraction and re-identification more challenging. Although an increased number of records used in the encoding process requires additional computation time, the simulation results confirmed that it can demonstrate strong resilience against re-identification attacks. Our approach of minimizing the relationship between source and encoded records to preserve privacy experimentally proved to be effective in defending against re-identification attacks.

Simulation Setup

This study simulates a scenario in which an attacker attempts to re-identify anonymized records using a plain data set. All simulations were performed on Ubuntu 20.04.1 with an Intel(R) Xeon(R) W-3245 3.20 GHz CPU and 256 GB of memory. Two datasets were used in the security durability evaluation simulation against re-identification attacks: the Titanic[1] and North Carolina Voter Registration (NCVR) databases[2]. In the Titanic dataset, we used attributes such as the first and last names of 1,317 passengers for encoding. The NCVR dataset, which was created in June 2014, was used in the simulation as both encoding and plain data for re-identification. This dataset includes information on 224,073 voters from North Carolina and comprises 19 attribute fields. However, the NCVR dataset contains numerous null values. Using these null values in the anonymization process can introduce noise or errors into the results. Therefore, to ensure an accurate simulation, we used information from 4,401 voters that did not contain any null values. In the simulation using the NCVR dataset, we randomly selected attributes for encoding from a pool of 17, excluding the two attributes with the highest and lowest cardinality values from a total of 19 user attributes.

Performance Evaluation

To evaluate the security performance of the proposed method, we measure the ratio of re-identified records. We also compare the ratio of re-identified records of the proposed scheme with the existing encoding methods, such as Bloom filter [19], Two-step hash [20], and Tabulation min-hash [30]. The results of the re-identification attack simulations for each dataset are presented in Fig ?? and Table 2. The primary metric of the evaluation, the ratio of re-identified records, was markedly lower with the zero-relationship method than with the other three encoding algorithms.

The simulation results using the Titanic dataset shown in Fig ?? confirmed the different security performances depending on the encoding algorithm. In Fig ??, the x-axis represents the matching algorithms (symmetric, Hungarian, Shapley) used to compare the similarity between the plain data and the data encoded by each algorithm. The y-axis indicates the ratio of re-identified records. Using the bloom filter, 1149, 1139, and 1150 records were re-identified for the Symmetric, Hungarian, and Shapley matching algorithms, respectively. In the case of the two-step hash, 1251 records were re-identified across all matching algorithms. The tabulation min-hash resulted in 658, 674, and 715 re-identified records, whereas the proposed zero-relationship method had only 18, 14, and 18 re-identified records. A similar trend was observed in the simulation results for the NCVR dataset in Table 2. The bloom filter resulted in 3993, 3999, and 3996 re-identified records. For the two-step hash, the re-identification counts were 4327,

[1] https://en.wikipedia.org/wiki/Passengers_of_the_Titanic
[2] http://dl.ncsbe.gov/

Table 2. Re-identification rates in NCVR dataset: Comparing encoding methods and matching algorithms.

Encoding method	Match Algorithms		
	Symmetric	Hungarian	Shapley
Bloom filter [19]	87.24 %	86.48 %	87.31 %
Two-step hash [20]	94.98 %	94.98 %	94.98 %
Tabulation min-hash [30]	49.96 %	51.17 %	54.29 %
Proposed	0 %	0 %	0 %

4. Results: Evaluation and Discussion

4.1. Simulation Setup

The performance of the proposed encoding method was assessed through experiments utilizing a re-identification attack simulator provided by [22]. The simulator, implemented in Python 2.7, was executed on a PC with a 64-bit Intel(R) Xeon(R) W-3245 3.20 GHz CPU and 256 GB of memory, running Ubuntu 20.04.1. Access to the utilized simulator is available via the link provided by the authors at https://dmm.anu.edu.au/pprlattack/. For the simulations, two types of public datasets were employed: the Titanic dataset [47] and the North Carolina Voter Registration (NCVR) database [48]. The Titanic dataset utilized attributes such as the first and last names of 1,317 passengers. The NCVR dataset (accessed in June 2014) contains information on 224,073 North Carolina voters across 19 attribute fields. Due to numerous null values across fields, which could introduce noise or errors if used without preprocessing, a subset of 4,401 voter records without any null values was employed for accurate simulation. In the simulations based on the NCVR dataset, attributes with the highest and lowest cardinality values were excluded, and a subset of the remaining 17 attributes was randomly selected for use.

4.2. Performance Evaluation

This section evaluates the proposed zero-relationship encoding method through a graph-based re-identification attack simulator. Our assessment focused on the method's effectiveness in preserving privacy by measuring the ratio of re-identified records. The encoding methods included in our comparison are the Bloom filter [36], Two-step hash [24], and Tabulation min-hash [37], all representing established privacy preservation techniques.

The experiments utilized two distinct datasets to conduct the evaluations: the Titanic and NCVR datasets, the results of which are illustrated in Figures 3 and 4, respectively. We observed that the zero-relationship encoding method significantly reduced the ratio of re-identified records across all matching algorithms compared to other encoding methods. The simulation results demonstrate that our encoding method effectively counters re-identification attacks by severing the explicit relationship between records.

The experiments utilizing the Titanic dataset demonstrated that traditional encoding methods like the Bloom filter, Two-step hash, and Tabulation min-hash resulted in a significant proportion of re-identified records. On average, these methods experienced re-identification rates of approximately 87%, 94.98%, and 50%, respectively, across various matching algorithms. In stark contrast, the proposed zero-relationship encoding method exhibited a remarkably lower average re-identification rate of around 1.3%, underscoring its superior performance in preserving privacy.

The NCVR dataset provided further evidence of the proposed method's robustness. The Bloom filter re-identified over 3,990 records for each matching algorithm, while the Two-step hash method showed a near-complete re-identification rate. The Tabulation min-hash offered an improved outcome yet allowed more than 1700 records to be re-identified. Strikingly, the proposed method achieved a zero re-identification rate, demonstrating an outstanding defense against re-identification attacks.

The proposed zero-relationship encoding method eliminates any linkage between the source and the encoded records, thwarting re-identification attempts. It employs a novel anonymization process that leverages the attributes of the source record to produce an outcome with minimal ties to the original data, thus significantly enhancing privacy preservation. Our method utilizes cardinality to prioritize the security of attributes. We exclude those with extremely high or low values to balance utility and security. The min-hash function is employed to obtain the smallest hash value for selected attributes, combined using XOR operations. This process not only enhances the protection of the dataset by transforming attribute data but also denies adversaries by obscuring the original information. This approach introduces uncertainty in identifying and classifying anonymized entities, significantly impairing the efficacy of potential attacks.

Our study further examined the relationship between the number of attributes used in the encoding process and the performance of the proposed method. Fig 5 shows the results of a performance measurement experiment of the unrelated encoding method depending on the number of attributes used for encoding. The NCVR dataset, which used three attributes, exhibited a lower re-identification rate than the Titanic dataset with two attributes. The graph demonstrates that as the number of attributes increases, there is a notable impact on both encoding and feature generation times. Specifically, a substantial rise in time required is observed as we move from 9 to 14 attributes. Despite this increase in computational effort, the associated rise in re-identification time indicates an enhancement in privacy protection. This trade-off highlights the method's scalability and potential to bolster privacy defenses with increased attribute counts, as higher complexity leads to reduced re-identification success. This nuanced understanding of performance dynamics is crucial for implementing the method in practical scenarios where attribute quantity and processing capabilities must be balanced.

The experiments conducted underscore the effectiveness of the proposed zero-relationship encoding method in mitigating the risk of data re-identification. Minimizing the relationship between the source and the encoded records provides a promising approach to privacy preservation in PPRL.

[Comment 1-6]
How are you justify the equation 1-2.

(Response 1-6)

We appreciate the reviewers' valuable comments.

The content within Section 3.1, "Problem Formulation," has been revised in response to the comments. The explanation has been strengthened to clarify and justify each equation.

The details of the revisions are as follows:

(Revision 1-6)

Before	After
[Section 3.1, p.4-5]<br>A re-identification attack is a particularly important security threat in a PPRL system and is defined as an attempt by an attacker to forcibly infer the source record from the anonymized record without the party's consent. The attacker encodes the entity within their database to maximize the probability that the encoded entity matches the anonymized entity of the others. The above problem can be formulated as follows:<br>$$\underset{e_n \in DB_p}{\text{argmax}}\ \Pr(\Xi(Encode(e_n), e^\tau) = M) \quad (1)$$<br>In Eq (1), the attacker attempts to find an entity e_n that matches e^τ, striving to maximize the probability that $\Xi(Encode(e_n), e^\tau)$ is classified as a match (M). However, inferring the original entity from the anonymized entity is challenging; therefore, the attacker can increase the success rate of re-identification by leveraging the frequency of specific attributes or known auxiliary information.<br>The primary goal of this research is to prevent privacy breaches due to re-identification attacks. Therefore, we attempt to reduce the success rate of re-identification attacks using an encoding method that is resistant to re-identification attacks. The main goal can be formulated as follows:<br>$$\underset{Encode}{\text{argmin}}\ \underset{e_n \in DB_p}{\text{argmax}}\ \Pr(\Xi(Encode(e_n), e^\tau) = M) \quad (2)$$<br>Eq (2) minimizes Eq (1) using a robust encoding method *Encode* that is resilient to re-identification attacks. *Encode* represents the encoding scheme of a record, and the maximum probability is calculated for every entity e_n within DB_p. Using this approach, we determine the encoding methods that best reduce the success rate of re-identification attacks.	***[Section 3.1, p.5-6]***<br>A re-identification attack, defined as an attacker's attempt to deduce the source record from an anonymized record without a permit, poses a significant threat to the security of a PPRL system. Attackers execute the record-matching process using datasets for re-identification attacks. They aim to identify records that bear a similar anonymized form to the anonymized record. To increase the success rate of their re-identification efforts, attackers seek records with a high probability of matching. This challenge is formulated as follows:<br>$$\underset{e_n \in DB_p}{\text{argmax}}\ \Pr(\Xi(\text{Encode}(e_n), e^\tau) = M) \quad (1)$$<br>Equation 1 delineates the process within a PPRL system of identifying a record within the database DB_p that maximizes the probability of a match with a given anonymized record e^τ. This models the attacker's scenario of attempting re-identification to locate the source record. The expression $\Pr(\Xi(\text{Encode}(e_n), e^\tau) = M)$ signifies the probability of deriving e^τ from e_n. Ξ is the decision model previously defined, tasked with determining whether a pair of anonymized records refer to the same entity. However, the inference of e_n from e^τ presents considerable difficulty, with the attacker's objective being to identify the e_n that maximizes the probability of a match. This entails an exhaustive consideration of all potential e_ns to select the record with the highest probability of correlation.<br>The paramount goal of this research is to preserve privacy breaches stemming from re-identification attacks. Thus, we strive to diminish the success rate of such attacks by employing an encoding method impervious to re-identification threats. This principal objective is mathematically formulated as follows:<br>$$\underset{\text{Encode}}{\text{argmin}}\ \underset{e_n \in DB_p}{\text{argmax}}\ \Pr(\Xi(\text{Encode}(e_n), e^\tau) = M) \quad (2)$$<br>Equation 2 seeks an encoding method that minimizes the probability established in Equation 1. The argmax component represents the attacker's endeavor to pinpoint the e_n that ensures the maximum probability of a match. Conversely, the argmin aspect indicates the quest for an encoding method that minimizes this match probability. Equation 2 accentuates how the encoding method can bolster resistance to re-identification attacks. An efficacious encoding method minimizes the linkage between e_n and e^τ, thereby reducing the likelihood of an attacker successfully deducing the source record. Consequently, Equation 2 is instrumental in determining encoding methods adept at countering re-identification attacks.

[Comment 1-7]

Encoding and decoding is old techniques, why are you using this?

(Response 1-7)

We appreciate the reviewers' valuable comments.

In response to Comment 1-7 regarding our use of encoding and decoding techniques, we understand the concerns regarding their perceived antiquity. However, we wish to clarify the rationale behind our choice, particularly in the context of Privacy-Preserving Record Linkage (PPRL).

Encoding is crucial in PPRL due to the necessity of handling massive datasets, such as medical records, where traditional encryption methods prove inefficient. Unlike standard encryption, encoding allows for the calculation of similarities between records, enabling the processing of anonymized data efficiently. While homomorphic encryption and searchable encryption can perform similar operations, they are significantly less efficient for tasks requiring similarity calculations, which are essential in record linkage.

Given the unique demands of PPRL, where efficiency and the ability to perform record linkage under anonymization are prioritized, the use of encoding techniques is justified despite a slight trade-off in security. This approach is not only appropriate but preferred for RL tasks due to its balance between security and operational efficiency.

To enhance the credibility of our methodology and address the reviewer's concerns, we added references that underscore the efficiency and suitability of encoding techniques for PPRL scenarios. These references will help provide a solid foundation for our choice and demonstrate its relevance and necessity in the context of our research.

The details of the revisions are as follows:

(Revision 1-7)

Before	After
[-]	***[Reference, p.13-16]***<br>[38] Gkoulalas-Divanis A, Vatsalan D, Karapiperis D, Kantarcioglu M. Modern privacy-preserving record linkage techniques: An overview. IEEE Transactions on Information Forensics and Security. 2021;.

[Comment 1-8]

Elaborate the result section with using of proper results. This paper is like a review paper.

(Response 1-8)

We appreciate the reviewers' valuable comments.

In response to your comments, we have revised this section to include a detailed analysis of the experimental data. The section now effectively elaborates on the empirical results obtained from our simulations, providing a critical discussion of these findings' implications in the privacy-preserving record linkage context. By incorporating precise data points and comparative analysis, we ensure that our results substantiate the proposed method's performance, offering tangible evidence of its effectiveness and practicality. We believe these enhancements significantly improve the manuscript by providing the reader with a clear understanding of our research's validity and its contributions to the field.

The details of the revisions are as follows:

(Revision 1-5)

Before	After
[Section 4, p.7-8]<br>**Results: Evaluation and Discussion**<br>This section evaluates the privacy-preserving performance and feasibility of the proposed zero-relationship encoding method by simulating the re-identification attack outlined in [18].	***[Section 4, p.9-10]***

4330, and 4328 records, indicating that most of the records were re-identified. The tabulation min-hash showed improved performance with 1786, 1768, and 1802 re-identified records. However, the proposed zero-relationship method demonstrated complete resistance to re-identification, with zero re-identified records for all matching algorithms. These results indicate that the zero-relationship encoding method offers excellent resistance to re-identification attacks by minimizing the risk of re-identification. Simulation results confirmed that the durability of the zero-relationship encoding method against re-identification attacks increased as the number of attributes used in the encoding process increased. We observed a lower re-identification rate in the experimental results of the NCVR dataset, which used three attributes, compared to the Titanic dataset, which used two attributes. Building on this finding, we designed experiments to quantitatively assess the impact of attribute count on performance metrics such as encoding time, feature extraction time, and re-identification attack time. Fig **??** shows the durability of the zero-relationship encoding method against re-identification attacks depending on the number of attributes. We verified that the increase in the number of attributes used in the encoding process increases the time spent on encoding. The time required for feature extraction and re-identification attacks also increased. Particularly when more than 14 attributes were used with encoding, we observed a sharp increase in the time required. This suggests that using numerous attributes for encoding results in the creation of a form without specific meaning, making feature extraction and re-identification more challenging. Although an increased number of records used in the encoding process requires additional computation time, the simulation results confirmed that it can demonstrate strong resilience against re-identification attacks. Our approach of minimizing the relationship between source and encoded records to preserve privacy experimentally proved to be effective in defending against re-identification attacks.

Simulation Setup

This study simulates a scenario in which an attacker attempts to re-identify anonymized records using a plain data set. All simulations were performed on Ubuntu 20.04.1 with an Intel(R) Xeon(R) W-3245 3.20 GHz CPU and 256 GB of memory. Two datasets were used in the security durability evaluation simulation against re-identification attacks: the Titanic[1] and North Carolina Voter Registration (NCVR) databases[2]. In the Titanic dataset, we used attributes such as the first and last names of 1,317 passengers for encoding. The NCVR dataset, which was created in June 2014, was used in the simulation as both encoding and plain data for re-identification. This dataset includes information on 224,073 voters from North Carolina and comprises 19 attribute fields. However, the NCVR dataset contains numerous null values. Using these null values in the anonymization process can introduce noise or errors into the results. Therefore, to ensure an accurate simulation, we used information from 4,401 voters that did not contain any null values. In the simulation using the NCVR dataset, we randomly selected attributes for encoding from a pool of 17, excluding the two attributes with the highest and lowest cardinality values from a total of 19 user attributes.

Performance Evaluation

To evaluate the security performance of the proposed method, we measure the ratio of re-identified records. We also compare the ratio of re-identified records of the proposed scheme with the existing encoding methods, such as Bloom filter [19], Two-step hash [20], and Tabulation min-hash [30]. The results of the re-identification attack simulations for each dataset are presented in Fig **??** and Table 2. The primary metric of the evaluation, the ratio of re-identified records, was markedly lower with the zero-relationship method than with the other three encoding algorithms.

The simulation results using the Titanic dataset shown in Fig **??** confirmed the different security performances depending on the encoding algorithm. In Fig **??**, the x-axis represents the matching algorithms (symmetric, Hungarian, Shapley) used to compare the similarity between the plain data and the data encoded by each algorithm. The y-axis indicates the ratio of re-identified records. Using the bloom filter, 1149, 1139, and 1150 records were re-identified for the Symmetric, Hungarian, and Shapley matching algorithms, respectively. In the case of the two-step hash, 1251 records were re-identified across all matching algorithms. The tabulation min-hash resulted in 658, 674, and 715 re-identified records, whereas the proposed zero-relationship method had only 18, 14, and 18 re-identified records. A similar trend was observed in the simulation results for the NCVR dataset in Table 2. The bloom filter resulted in 3993, 3999, and 3996 re-identified records. For the two-step hash, the re-identification counts were 4327,

[1] https://en.wikipedia.org/wiki/Passengers_of_the_Titanic
[2] http://dl.ncsbe.gov/

Table 2. Re-identification rates in NCVR dataset: Comparing encoding methods and matching algorithms.

Encoding method	Match Algorithms		
	Symmetric	Hungarian	Shapley
Bloom filter [19]	87.24 %	86.48 %	87.31 %
Two-step hash [20]	94.98 %	94.98 %	94.98 %
Tabulation min-hash [30]	49.96 %	51.17 %	54.29 %
Proposed	0 %	0 %	0 %

4. Results: Evaluation and Discussion

4.1. Simulation Setup

The performance of the proposed encoding method was assessed through experiments utilizing a re-identification attack simulator provided by [22]. The simulator, implemented in Python 2.7, was executed on a PC with a 64-bit Intel(R) Xeon(R) W-3245 3.20 GHz CPU and 256 GB of memory, running Ubuntu 20.04.1. Access to the utilized simulator is available via the link provided by the authors at https://dmm.anu.edu.au/pprlattack/. For the simulations, two types of public datasets were employed: the Titanic dataset [47] and the North Carolina Voter Registration (NCVR) database [48]. The Titanic dataset utilized attributes such as the first and last names of 1,317 passengers. The NCVR dataset (accessed in June 2014) contains information on 224,073 North Carolina voters across 19 attribute fields. Due to numerous null values across fields, which could introduce noise or errors if used without preprocessing, a subset of 4,401 voter records without any null values was employed for accurate simulation. In the simulations based on the NCVR dataset, attributes with the highest and lowest cardinality values were excluded, and a subset of the remaining 17 attributes was randomly selected for use.

4.2. Performance Evaluation

This section evaluates the proposed zero-relationship encoding method through a graph-based re-identification attack simulator. Our assessment focused on the method's effectiveness in preserving privacy by measuring the ratio of re-identified records. The encoding methods included in our comparison are the Bloom filter [36], Two-step hash [24], and Tabulation min-hash [37], all representing established privacy preservation techniques.

The experiments utilized two distinct datasets to conduct the evaluations: the Titanic and NCVR datasets, the results of which are illustrated in Figures 3 and 4, respectively. We observed that the zero-relationship encoding method significantly reduced the ratio of re-identified records across all matching algorithms compared to other encoding methods. The simulation results demonstrate that our encoding method effectively counters re-identification attacks by severing the explicit relationship between records.

The experiments utilizing the Titanic dataset demonstrated that traditional encoding methods like the Bloom filter, Two-step hash, and Tabulation min-hash resulted in a significant proportion of re-identified records. On average, these methods experienced re-identification rates of approximately 87%, 94.98%, and 50%, respectively, across various matching algorithms. In stark contrast, the proposed zero-relationship encoding method exhibited a remarkably lower average re-identification rate of around 1.3%, underscoring its superior performance in preserving privacy.

The NCVR dataset provided further evidence of the proposed method's robustness. The Bloom filter re-identified over 3,990 records for each matching algorithm, while the Two-step hash method showed a near-complete re-identification rate. The Tabulation min-hash offered an improved outcome yet allowed more than 1700 records to be re-identified. Strikingly, the proposed method achieved a zero re-identification rate, demonstrating an outstanding defense against re-identification attacks.

The proposed zero-relationship encoding method eliminates any linkage between the source and the encoded records, thwarting re-identification attempts. It employs a novel anonymization process that leverages the attributes of the source record to produce an outcome with minimal ties to the original data, thus significantly enhancing privacy preservation. Our method utilizes cardinality to prioritize the security of attributes. We exclude those with extremely high or low values to balance utility and security. The min-hash function is employed to obtain the smallest hash value for selected attributes, combined using XOR operations. This process not only enhances the protection of the dataset by transforming attribute data but also denies adversaries by obscuring the original information. This approach introduces uncertainty in identifying and classifying anonymized entities, significantly impairing the efficacy of potential attacks.

Our study further examined the relationship between the number of attributes used in the encoding process and the performance of the proposed method. Fig 5 shows the results of a performance measurement experiment of the unrelated encoding method depending on the number of attributes used for encoding. The NCVR dataset, which used three attributes, exhibited a lower re-identification rate than the Titanic dataset with two attributes. The graph demonstrates that as the number of attributes increases, there is a notable impact on both encoding and feature generation times. Specifically, a substantial rise in time required is observed as we move from 9 to 14 attributes. Despite this increase in computational effort, the associated rise in re-identification time indicates an enhancement in privacy protection. This trade-off highlights the method's scalability and potential to bolster privacy defenses with increased attribute counts, as higher complexity leads to reduced re-identification success. This nuanced understanding of performance dynamics is crucial for implementing the method in practical scenarios where attribute quantity and processing capabilities must be balanced.

The experiments conducted underscore the effectiveness of the proposed zero-relationship encoding method in mitigating the risk of data re-identification. Minimizing the relationship between the source and the encoded records provides a promising approach to privacy preservation in PPRL.

Figure 3. Re-identification rates in the Titanic dataset: Comparing encoding methods and matching algorithms.

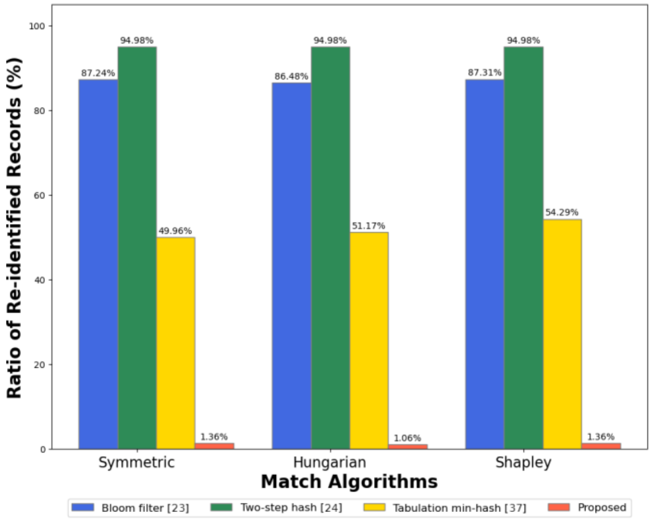

Encoding method	Match Algorithms		
	Symmetric	Hungarian	Shapley
Bloom filter [23]	87.24 % (1149)	86.48 % (1139)	87.31 % (1150)
Two-step hash [24]	94.98 % (1251)	94.98 % (1251)	94.98 % (1251)
Tabulation min-hash [37]	49.96 % (658)	51.17 % (674)	54.29 % (715)
Proposed scheme	1.36 % (18)	1.06 % (14)	1.36 % (18)

Figure 4. Re-identification rates in the NCVR dataset: Comparing encoding methods and matching algorithms.

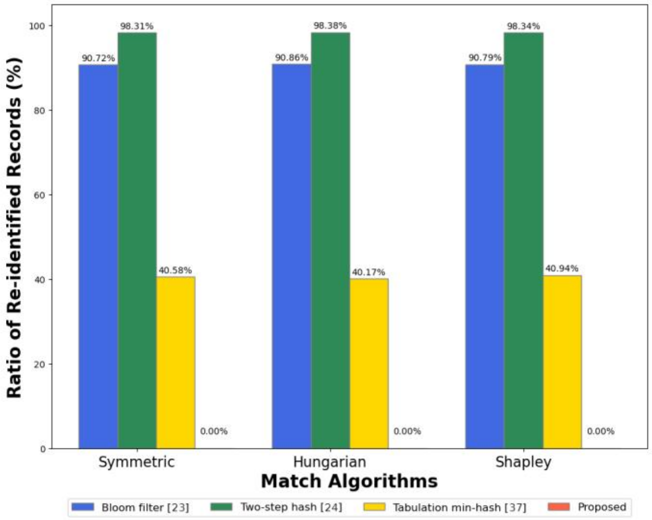

Encoding method	Match Algorithms		
	Symmetric	Hungarian	Shapley
Bloom filter [23]	90.72 % (3993)	90.86 % (3999)	90.79 % (3996)
Two-step hash [24]	98.31 % (4327)	98.38 % (4330)	98.34 % (4328)
Tabulation min-hash [37]	40.58 % (1786)	40.17 % (1768)	40.94 % (1802)
Proposed scheme	0 % (0)	0 % (0)	0 % (0)

Figure 5. Performance of the zero-relationship encoding method according to the number of attributes used for encod-
ing

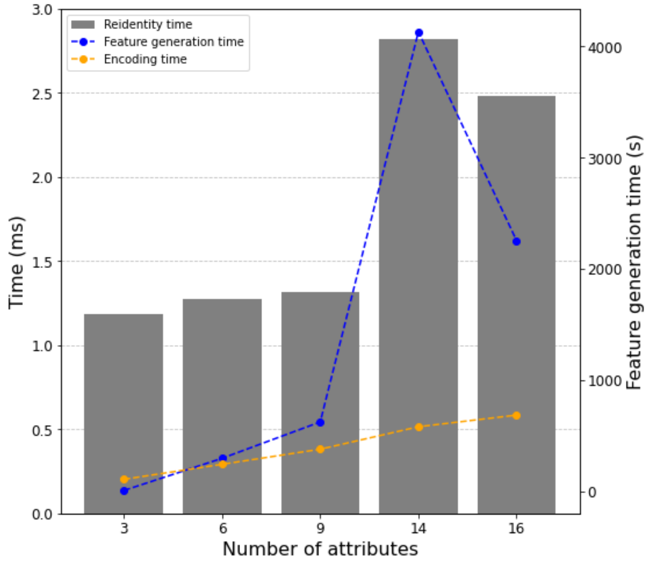

– Thank you so much –

[Response to Reviewer 2 Comments]

[Comment 2-1]
Please improve the Abstract.

(Response 2-1)
We appreciate the reviewers' valuable comments.

In response to comments 2-1 regarding the abstract section, we have revised the abstract to be concise and clearer.

The details of the revisions are as follows:

(Revision 2-1)

Before	After
[Abstract, p.1]<br>Recent studies have revealed that privacy-preserving record linkage (PPRL) technology is vulnerable to graph-based re-identification attacks. This poses a severe security issue for PPRL, which links similar records across multiple data sets while preserving privacy. Graph-based re-identification attack techniques infer source records by contrasting plain text graphs with encoded record graphs, which can neutralize the privacy-preserving technology of PRRL and steal privacy. Leaked privacy can be exploited for crimes, such as identity theft and financial fraud, causing severe harm to people. Therefore, a new PPRL technology that can effectively resist re-identification attacks and securely preserve user privacy is necessary. This study proposes a zero-relationship encoding scheme to prevent record re-identification attacks in PPRL systems. The proposed zero-relationship encoding method minimizes the relationship between the source and encoded records, preventing an attacker from inferring the source record. To verify the privacy-preserving performance of the proposed method, a record re-identification attack simulation was performed using the Titanic and North Carolina Voter Records (NCVR) datasets. Simulation results show that an average of 16 (1.21\%) of the 1317 records in the Titanic data set were re-identified. For the NCVR dataset, all 4401 records resisted the	***[Abstract, p.1]***<br>Privacy-preserving record linkage (PPRL) technology, crucial for linking records across datasets while maintaining privacy, is susceptible to graph-based re-identification attacks. These attacks compromise privacy and pose significant risks, such as identity theft and financial fraud. This study introduces a novel zero-relationship encoding scheme designed to enhance PPRL systems' resistance to re-identification attacks by minimizing the linkage between source and encoded records. Our method's efficacy was validated through simulations on the Titanic and North Carolina Voter Records (NCVR) datasets, demonstrating a substantial reduction in re-identification rates. Security analysis confirms that our zero-relationship encoding effectively preserves privacy against graph-based re-identification threats, improving PPRL technology's security.

re-identification attack, achieving a re-identification rate of 0\%. Security analysis proves zero-relationship encoding preserves user privacy by defending against graph-based re-identification attacks.	

[Comment 2-2]
The Introduction section is very poor. In a research article, the introduction section must be very strong with the motivations of this paper, which is missing in this paper. Moreover, the disadvantages of the existing schemes must be discussed to motivate this new work.

(Response 2-2)
We appreciate the reviewers' valuable comments.

In response to the feedback on the Introduction section, we have made significant improvements to enhance its strength and clarity. The revised introduction now effectively highlights the motivations behind our research and clearly articulates the need for our proposed approach.

The details of the revisions are as follows:

(Revision 2-2)

Before	After
[Section 1, p.2]<br>**1. Introduction**<br>The increase in user data collection for personalized services in various applications such as media, advertising, and healthcare, has intensified concerns over privacy preservation [1–5]. These privacy issues become more severe when integrated data are created by linking records from diverse sources to improve data quality and achieve accurate analysis results [6,7]. Record linkage is primarily performed using quasi-identifiers (QIDs), which are attributes that can be used for identification, such as names, addresses, and dates of birth [8,9]. This approach enables accurate record linkage because it uses information directly associated with the entities [10]. This approach poses security issues when information is leaked to hackers, thereby allowing them to identify entities for criminal targeting or misuse [11]. Therefore, many organizations are currently utilizing privacy-preserving record linkage (PPRL) technology to securely link records from different data sources [12].	***[Section 1, p.2]***<br>**1. Introduction**<br>The escalating collection of user data for personalized services across various sectors in media advertising, the Internet of things, and healthcare has heightened privacy concerns significantly [1–6]. Integrating data from diverse sources through record linkage exacerbates these privacy challenges [7,8], especially when personally identifiable information (PII) like name, email address, or passport number is employed for linkage [9–11]. Despite its high accuracy, this conventional approach poses substantial security risks by potentially exposing individuals to identity theft and misuse of information [12–14].<br>In response to these vulnerabilities, Privacy-Preserving Record Linkage (PPRL) technologies have been developed to link records across different datasets while privacy preservation securely [15–18]. However, PPRL's effectiveness is undermined by sophisticated graph-based re-identification attacks [19–21]. These attacks can decrypt anonymized records, thus nullifying PPRL's privacy-preserving capabilities [22]. The gravity of these attacks cannot be overstated, as they can lead to severe consequences, including identity theft and financial fraud. Fig 1 presents a simplified overview of the graph-based re-identification attack proposed in [22].<br>Although innovative, existing solutions, such as split bloom filters and two-step hash methods, fall short of fully safeguarding against these re-identification attacks [23–26]. These methods do not adequately address the inherent relationship between source and anonymized records, leaving a window for potential privacy breaches [27].

<table>
<tr>
<td>
PPRL is a record linkage technology that preserves privacy by anonymizing information from different data sources and subsequently calculating and linking matching records. Comparing and accurately linking anonymized records is not straightforward because of typographical errors, modifications, and duplications. Therefore, in PPRL, the similarity of anonymized record pairs is calculated, and record pairs above a user-set threshold are linked. The PPRL technology, which provides high flexibility, is used in various fields for data linkage by allowing a certain level of errors [13–15].
However, recent research has revealed a security vulnerability in PPRL, in which anonymized records can be re-identified to locate the original data [16,17]. The graph-based re-identification attack proposed by Vidanage et al. uses specific patterns that arise when data are mapped into q-grams to anonymize records in PPRL [18]. A graph is generated using the encoded data from PPRL to extract the features of the pattern. The extracted features are subsequently used to locate the plain text of the encoded data through a comparative analysis with a graph based on plain data. Fig 1 presents a simplified overview of the graph-based re-identification attack proposed in [18]. The re-identification of anonymized records threatens the goal of PPRL to preserve privacy. Therefore, it is necessary to develop robust security methods to address this issue. New approaches to prevent re-identification have been studied, such as a split bloom filter method that minimizes the exposure of anonymized data and a two-step hash method that hashes records twice [19,20]. Nonetheless, residual relationships can still exist between the source and the anonymized records, enabling re-identification attacks [18]. Therefore, research on an anonymization method that considers the relationship between the source and the anonymized records is needed to prevent re-identification attacks. Moreover, there is a need to develop a secure PPRL framework that applies these anonymization techniques.
This study proposes a zero-relationship encoding method to prevent privacy leakage threats from graph-based re-identification attacks in PPRL systems. The proposed encoding method effectively defends against re-identification attacks by minimizing the relationship between the source records and the encoded results. The main contributions of this study are as follows:

We demonstrate that the anonymization techniques currently used in PPRL do not consider the relationship between the source and the anonymized results, making them susceptible to graph-based re-identification attacks.
We propose the concept of data relatedness in PPRL, which should be considered when anonymizing data to preserve privacy. This concept addresses the relationship between source data and anonymized data.
We propose a zero-relationship encoding method that minimizes the relationship between source and encoded data, preventing re-identification attacks based on brute force.
We provide an intuitive indicator to evaluate the resistance of each encoding method against graph-based re-identification attacks.

The remainder of this paper is organized as follows: The Methods section details the proposed zero-relationship encoding method and demonstrates that the PPRL system is safe from re-identification attacks. In the Results section, we evaluate the performance of the proposed method and compare it with related studies. Finally, the Conclusion section concludes the paper.
</td>
<td>
This study proposes a novel zero-relationship encoding scheme to fortify PPRL's security against graph-based re-identification attacks. Our method significantly diminishes the risk of privacy breaches by effectively minimizing the relationship between source and encoded records. We empirically validate the efficacy of our approach through simulations using the Titanic and North Carolina Voter Records (NCVR) datasets, demonstrating superior resistance to re-identification attacks compared to existing methodologies. The main contributions of this study are as follows:

We propose a zero-relationship encoding scheme to defend against re-identification attacks within PPRL systems. Unlike traditional anonymization techniques, our method ensures minimal relational links between the source and encoded records, significantly enhancing privacy preservation.
We propose a novel PPRL framework incorporating the zero-relationship encoding method. This framework is tailored to resist graph-based re-identification attacks, addressing a critical security vulnerability in existing PPRL technologies.
Through comprehensive simulations using real-world datasets (Titanic and North Carolina Voter Records), we demonstrate the superior resistance of our proposed method against re-identification attacks. The proposed methods significantly reduce re-identification rates compared to existing techniques.
We introduce a concrete method for evaluating the effectiveness of different encoding methods in preserving privacy and resisting re-identification attacks. We utilize entropy and information gain as metrics to assess the security performance of the encoding method quantitatively. This contribution provides a practical tool for researchers and practitioners to evaluate the security efficacy of their PPRL solutions.

Our research fills a security vulnerability in PPRL by providing a robust solution to the re-identification attacks. It underscores the necessity for encoding methods that consider the relationship between source and anonymized records, offering a blueprint for future developments in secure PPRL frameworks.
The remainder of this paper is organized as follows: The privacy-preservation study to improve PPRL's security section introduces related studies. The Methods section details the proposed zero-relationship encoding method and demonstrates that the PPRL system is safe from re-identification attacks. We evaluate the proposed method's performance in the Results section and compare it with related studies. Finally, the Conclusion section concludes the paper.
</td>
</tr>
</table>

[Comment 2-3]

The point-wise contributions mentioned in the Introduction section are not specific.

(Response 2-3)

We appreciate the reviewers' valuable comments.

In response to your comment, we have thoroughly revised the contributions section to ensure that each point is specific, direct, and reflective of the novel aspects of our research.

The details of the revisions are as follows:

(Revision 2-3)

Before	After

[Section 1, p.2]	***[Section 1, p.2]***
• We demonstrate that the anonymization techniques currently used in PPRL do not consider the relationship between the source and the anonymized results, making them susceptible to graph-based re-identification attacks.<br>• We propose the concept of data relatedness in PPRL, which should be considered when anonymizing data to preserve privacy. This concept addresses the relationship between source data and anonymized data.<br>• We propose a zero-relationship encoding method that minimizes the relationship between source and encoded data, preventing re-identification attacks based on brute force.<br>• We provide an intuitive indicator to evaluate the resistance of each encoding method against graph-based re-identification attacks.	• We propose a zero-relationship encoding scheme to defend against re-identification attacks within PPRL systems. Unlike traditional anonymization techniques, our method ensures minimal relational links between the source and encoded records, significantly enhancing privacy preservation.<br>• We propose a novel PPRL framework incorporating the zero-relationship encoding method. This framework is tailored to resist graph-based re-identification attacks, addressing a critical security vulnerability in existing PPRL technologies.<br>• Through comprehensive simulations using real-world datasets (Titanic and North Carolina Voter Records), we demonstrate the superior resistance of our proposed method against re-identification attacks. The proposed methods significantly reduce re-identification rates compared to existing techniques.<br>• We introduce a concrete method for evaluating the effectiveness of different encoding methods in preserving privacy and resisting re-identification attacks. We utilize entropy and information gain as metrics to assess the security performance of the encoding method quantitatively. This contribution provides a practical tool for researchers and practitioners to evaluate the security efficacy of their PPRL solutions.

[Comment 2-4]

The Related Work section is poor. The authors must include some more recent schemes. Also, the following papers must be cited to improve this section, as well as the Reference section:

— Network anomaly detection using deep learning techniques
— Problem-based cybersecurity lab with knowledge graph as guidance
— Data accessing based on the popularity value for cloud computing
— Local binary pattern-based reversible data hiding
— Enhancing security of medical images using deep learning, chaotic map, and hash table

- Review on offloading of vehicle edge computing
- Security, privacy, trust, and anonymity

(Response 2-4)

We appreciate the reviewers' valuable comments.

In response to your feedback, we have taken the following actions to enhance the quality and comprehensiveness of our manuscript:

Comprehensive Literature Review: We conducted an extensive literature search to identify and review our field's most recent and relevant research. This effort ensured that our Related Work section reflected the latest developments and provided a thorough background for our study.

Inclusion of Recommended Papers: We carefully reviewed each of the seven papers you recommended and used them as references in the Related Work section and other sections.

Creation of a Summary Table: To better understand the recent studies and their relation to our work, we created a summary table. This table organizes the key findings, methodologies, and limitations of the recent research we reviewed. This addition will help readers quickly grasp the current research landscape and see how our work fits.

The details of the revisions are as follows:

(Revision 2-4)

Before	After
[Section 2, p.3-4]	***[Section 2, p.2-4]***<br>2. Privacy-preservation study to improve PPRL's security<br><br>In recent years, numerous security methods have been developed to address the security vulnerability in PPRL. These security methods are classified into five categories: cryptography [28,29], embedding techniques [30], differential privacy [31,32], statistical linkage key [33,34], and probabilistic methods [24,35–37]. Among these, probabilistic methods have garnered attention because of their computational efficiency and capability to manage large-scale datasets [38]. However, security methods based on probabilistic approaches are vulnerable to privacy attacks, such as re-identification, inference, man-in-the-middle, and dictionary attacks [21]. Privacy leaks due to privacy attacks are a severe problem that undermines the purpose of PPRL [25–27]. Therefore, various methods have been proposed to overcome these limitations. We introduce some studies that are representative of improving the security performance of PPRL.

2. Related Work

In recent years, numerous security methods have been developed to address the vulnerability issues in PPRL. These security methods are classified into five categories: cryptography [21,22], embedding techniques [23], differential privacy [24,25], statistical linkage key [26,27], and probabilistic methods [20,28–30]. Among these, probabilistic methods have garnered attention because of their computational efficiency and capability to manage large-scale datasets. However, security methods based on probabilistic approaches are vulnerable to privacy attacks, such as re-identification, inference, man-in-the-middle, and dictionary attacks. Privacy leaks due to privacy attacks are a serious problem that undermines the purpose of PPRL. Therefore, various methods have been proposed to overcome these limitations.

Ranbaduge *et al.* proposed a windowing-based XORing (WXOR) method to mitigate the vulnerability of the bloom filter encoding method [29]. The WXOR method employs a sliding window approach on the BF, searching for adjacent bit patterns, and subsequently XORs these patterns to generate a new one. The flexibility of adjusting the window size allows optimization in terms of encoding process efficiency or security enhancement. Another study introduced a new hash-based encoding method [20]. The proposed secure two-step hash encoding method hashes private record information into a bit matrix, with each column hashed to generate an integer set representation of a sensitive input value. Simulations using real data demonstrated that the method can substantially improve record linkage quality in large databases. However, both encoding methods are still vulnerable to re-identification attacks. Both approaches retain the characteristics of the source records in the encoded records, allowing the re-identification of records using features extracted through separate processing [12,31].

Smith proposed the tabulation min-hash (TMH) encoding method as a remedy to the vulnerabilities in the bloom filter encoding method [30]. The primary objective of this method is to safeguard privacy by circumventing the simple mapping of plaintexts encoded into q-grams. TMH utilizes a bit array of length l in tandem with a set of lookup tables to encode the records. Each set of lookup tables consists of multiple tables and keys. These keys, which symbolize random bit strings, are derived from the MinHash values of the q-gram set. These random bit strings undergo an XOR operation, resulting in a definitive bit string that is aggregated to form the final bit vector. Although TMH offers strong security performance owing to its high computational complexity, it remains vulnerable to re-identification attacks. This vulnerability stems from the approach of preserving the minimum number of bits for similarity comparison during encoding. These minimum bits retain the characteristics of the source record, enabling attackers to extract features from the encoded record and subsequently reidentify it. Therefore, TMH is unsafe against re-identification attacks.

Nóbrega *et al.* highlighted the shortcomings of existing PPRL research that assumed an unrealistic adversarial model and proposed an auditable blockchain-based PPRL (ABEL) model that considers a more realistic adversarial model [19]. Additionally, they proposed a splitting bloom filter (SBF) method to address the vulnerabilities inherent in the conventional bloom filter. This method minimizes the information shared between parties involved in the record linkage process. Unlike conventional methods that require the exchange of the entire bloom filter to check for similarity during the comparison stage of PPRL, the SBF method only exchanges portions of the original bloom filter. The method reduces the risk of data leakage by limiting the amount of shared information, thereby enhancing security. However, ABEL still presents security vulnerabilities. In the bloom filter segment exchange protocol proposed for database owners, a re-identification attack becomes feasible if a user receiving a bloom filter segment from another user turns malicious [32]. An attacker possessing the same encoding knowledge as a regular user can utilize their database to find a set of q-grams that can be hashed to that specific location in the segment, resulting in privacy breaches through the re-identification of records encoded by regular users.

Existing studies have overlooked the relationship between the source and encoded records during the encoding process. The closer the relationship between the source and encoded records, the easier it is to decrypt them. An attacker can exploit this advantage to acquire the information required to infer the source data from the encoded data. Recent studies have demonstrated that attackers can identify a source record by launching a dictionary attack on the encoded record, provided they have knowledge of the encoding parameters [17,18]. Therefore, developing a security method that considers the relationship between the source and encoded records is crucial during record encoding.

- Ranbaduge *et al.* proposed a windowing-based XORing (WXOR) method to mitigate the vulnerability of the bloom filter encoding method [36]. The WXOR method employs a sliding window approach on the bloom filter encoding (BF), searching for adjacent bit patterns, and subsequently XORs these patterns to generate a new one. The flexibility of adjusting the window size allows optimization in terms of encoding process efficiency or security enhancement. Another study introduced a new hash-based encoding method [24]. The proposed secure two-step hash encoding method hashes private record information into a bit matrix, with each column hashed to generate an integer set representation of a sensitive input value. Simulations using real data demonstrated that the method can substantially improve record linkage quality in large databases. However, both encoding methods are still vulnerable to re-identification attacks. Both approaches retain the characteristics of the source records in the encoded records, allowing the re-identification of records using features extracted through separate processing [39,40].

- Smith proposed the tabulation min-hash (TMH) encoding method as a remedy to the vulnerabilities in the bloom filter encoding method [37]. The primary objective of this method is to safeguard privacy by circumventing the simple mapping of plaintexts encoded into q-grams. TMH utilizes a bit array of length l in tandem with a set of lookup tables to encode the records. Each set of lookup tables consists of multiple tables and keys. These keys, which symbolize random bit strings, are derived from the MinHash values of the q-gram set. These random bit strings undergo an XOR operation, resulting in a definitive bit string aggregated to form the final bit vector. Although TMH offers strong security performance owing to its high computational complexity, it remains vulnerable to re-identification attacks. This vulnerability stems from the approach of preserving the minimum number of bits for similarity comparison during encoding. These minimum bits retain the characteristics of the source record, enabling attackers to extract features from the encoded record and subsequently re-identify it [?]detect). Therefore, TMH is unsafe against re-identification attacks.

- Nóbrega *et al.* highlighted the shortcomings of existing PPRL research that assumed an unrealistic adversarial model and proposed an auditable blockchain-based PPRL (ABEL) model that considers a more realistic adversarial model [23]. Additionally, they proposed a splitting bloom filter (SBF) method to address the vulnerabilities inherent in the conventional bloom filter. This method minimizes the information shared between parties involved in the record linkage process. Unlike traditional methods that require the exchange of the entire bloom filter to check for similarity during the comparison stage of PPRL, the SBF method only exchanges portions of the original bloom filter. The method reduces the risk of data leakage by limiting the amount of shared information, thereby enhancing security. However, ABEL still presents security vulnerabilities. In the bloom filter segment exchange protocol proposed for database owners, a re-identification attack becomes feasible if a user receiving a bloom filter segment from another user turns malicious [41,42]. An attacker possessing the same encoding knowledge as a regular user can utilize their database to find a set of q-grams that can be hashed to that specific location in the segment, resulting in privacy breaches through the re-identification of records encoded by regular users.

Existing studies have overlooked the relationship between the source and encoded records during the encoding process. The closer the relationship between the source and encoded records, the easier it is to decrypt them. An attacker can exploit this advantage to acquire the information required to infer the source data from the encoded data.

Table 1. Related work to improve the security of PPRL

Year	Author	Description	Limitation
2017	Smith D.	• Proposed tabulation min-hash (TMH) to remedy the BF encoding method vulnerabilities. Privacy preservation by avoiding simple mapping of plain-texts encoded into q-grams.<br>• Utilizes a bit array of length and lookup tables to encode records based on min-hash values of the q-gram set.	Maintaining essential bits for similarity in encoding.
2020	Ranbaduge et al.	• The windowing-based XORing (WXOR) method is proposed to mitigate vulnerabilities inherent in the BF encoding approach.<br>• Employs a sliding window to search for adjacent bit patterns within q-grams, subsequently combining these patterns using XOR to create new ones.<br>• The window size can be adjusted, allowing for pattern size alterations and optimizing the encoding process's efficiency and security.	Relationships between source and encoded records.
2020	Ranbaduge et al.	• Proposed a secure two-step hash encoding method. Hashes private record information into a bit matrix. Each column is hashed to generate an integer set representation of a sensitive input value.<br>• Real data simulations demonstrate improved record linkage quality in large databases.	Relationships between source and encoded records.
2021	Nóbrega et al.	• Assumed an unrealistic adversarial model and proposed an auditable blockchain-based PPRL (ABEL).<br>• Highlights existing PPRL research limitations that rely on unrealistic adversarial models.<br>• To minimize information exchange during the record linkage process, the splitting bloom filters (SBF) approach is proposed, which exchanges only parts of the encoded data, reducing security threats in the event of data breaches.	Re-identification risks arise if a user becomes malicious during BF exchange.
2022	Victor C. et al.	• Proposed an autoencoder-based method transforming BFs into numerical vectors, addressing vulnerabilities in BF encoding.<br>• Enhances security and privacy by preventing known BF attacks, offering a robust solution against re-identification risks.<br>• Data owners train autoencoder models for BF encoding/decoding, ensuring comparability via a mapping function.	Autoencoder sensitivity
2023	Siyu Y. et al.	• Proposed a PPRL using a Siamese neural network (SNN). SNN involves three main steps: combined bloom filter encoding module (CMF), blocking, and record matching using SNN. CMF combines a flat bloom filter (FBF) for strings and a transform bloom filter (TBF) for numerical attributes.<br>• Offers enhanced security via CMF encoding, precise record matching with SNN, adaptability to diverse data types, and robust performance in scenarios with data errors.	Scalability considerations and adaptation to data errors
2023	Frederik A. et al.	• Propose a BF with diffusion (BFD) PPRL scheme that approaches PPRL by extending conventional BFs with a linear diffusion layer.<br>• BFD enhances security against known attacks while preserving or improving linkage quality, potentially as a robust alternative to standard BF-based PPRL.	Complexity in balancing cost and fairness.
2024	Ours	• Proposes a zero-relationship encoding method to prevent re-identification attacks in PPRL and preserve privacy.<br>• Involves selecting quasi-identifiers based on their significance and applying XOR-folding to their hash values to generate encoded records, disrupting the re-identification process.<br>• Effectively protects privacy by preventing re-identification attacks and offers high efficiency due to low computational complexity in encoding.	Processing of records with a small number of attributes.

[Comment 2-5]
In section 2, a table can be given to summarize the entire section.

(Response 2-5)
We appreciate the reviewers' valuable comments.

In response to the comment in 2-5, we added a table summarizing the characteristics of relevant studies aiming to enhance the security of PPRL in the respective section of our paper. Table 1, titled "Related work to improve the security of PPRL," presents a summary of recent research on PPRL security in addition to the prominent studies introduced earlier, showcasing their summaries and limitations.

The details of the revisions are as follows:

(Revision 2-5)

**Before**	**After**
[-]	***[Section 2, p.4]***

Table 1. Related work to improve the security of PPRL

Year	Author	Description	Limitation
2017	Smith D.	• Proposed tabulation min-hash (TMH) to remedy the BF encoding method vulnerabilities. Privacy preservation by avoiding simple mapping of plaintexts encoded into q-grams.<br>• Utilizes a bit array of length and lookup tables to encode records based on min-hash values of the q-gram set.	Maintaining essential bits for similarity in encoding.
2020	Ranbaduge et al.	• The windowing-based XORing (WXOR) method is proposed to mitigate vulnerabilities inherent in the BF encoding approach.<br>• Employs a sliding window to search for adjacent bit patterns within q-grams, subsequently combining these patterns using XOR to create new ones.<br>• The window size can be adjusted, allowing for pattern size alterations and optimizing the encoding process's efficiency and security.	Relationships between source and encoded records.
2020	Ranbaduge et al.	• Proposed a secure two-step hash encoding method. Hashes private record information into a bit matrix. Each column is hashed to generate an integer set representation of a sensitive input value.<br>• Real data simulations demonstrate improved record linkage quality in large databases.	Relationships between source and encoded records.
2021	Nóbrega et al.	• Assumed an unrealistic adversarial model and proposed an auditable blockchain-based PPRL (ABEL).<br>• Highlights existing PPRL research limitations that rely on unrealistic adversarial models.<br>• To minimize information exchange during the record linkage process, the splitting bloom filters (SBF) approach is proposed, which exchanges only parts of the encoded data, reducing security threats in the event of data breaches.	Re-identification risks arise if a user becomes malicious during BF exchange.
2022	Victor C. et al.	• Proposed an autoencoder-based method transforming BFs into numerical vectors, addressing vulnerabilities in BF encoding.<br>• Enhances security and privacy by preventing known BF attacks, offering a robust solution against re-identification risks.<br>• Data owners train autoencoder models for BF encoding/decoding, ensuring comparability via a mapping function.	Autoencoder sensitivity
2023	Siyu Y. et al.	• Proposed a PPRL using a Siamese neural network (SNN). SNN involves three main steps: combined bloom filter encoding module (CMF), blocking, and record matching using SNN. CMF combines a flat bloom filter (FBF) for strings and a transform bloom filter (TBF) for numerical attributes.<br>• Offers enhanced security via CMF encoding, precise record matching with SNN, adaptability to diverse data types, and robust performance in scenarios with data errors.	Scalability considerations and adaptation to data errors
2023	Frederik A. et al.	• Propose a BF with diffusion (BFD) PPRL scheme that approaches PPRL by extending conventional BFs with a linear diffusion layer.<br>• BFD enhances security against known attacks while preserving or improving linkage quality, potentially as a robust alternative to standard BF-based PPRL.	Complexity in balancing cost and fairness.
2024	Ours	• Proposes a zero-relationship encoding method aimed at preventing re-identification attacks in PPRL and safeguarding privacy.<br>• Involves selecting quasi-identifiers based on their significance and applying XOR-folding to their hash values to generate encoded records, disrupting the re-identification process.<br>• Effectively protects privacy by preventing re-identification attacks and offers high efficiency due to low computational complexity in encoding.	Processing of records with a small number of attributes.

[Comment 2-6]
What is the use of the Zero-Relationship Encoding Method in the proposed scheme?

(Response 2-6)
We appreciate the reviewers' valuable comments.

The core function of the Zero-Relationship Encoding Method in our proposed scheme is to completely disrupt the relational ties between original records and their encoded counterparts for privacy-preserving record linkage. This method ingeniously eliminates any discernible link-

age patterns that could potentially facilitate graph-based re-identification attacks.

By employing a novel combination of hashing and XOR operations on selected quasi-identifiers, our method ensures that the encoded records bear no resemblance to the original data, making any attempt to reverse-engineer or match encoded records back to their source futile. This process is both effective and efficient, offering a robust solution to the critical challenge of safeguarding privacy in record linkage operations.

In essence, the Zero-Relationship Encoding Method provides a foundational layer of security by obfuscating the direct and indirect connections between data, thereby guaranteeing an enhanced level of privacy protection in our PPRL framework.

[Comment 2-7]
How the performance is increased?

(Response 2-7)
We appreciate the reviewers' valuable comments.

The performance of our proposed encoding scheme has been markedly improved over existing methods, as substantiated by our experimental outcomes.

Encoding method	Match Algorithms		
	Symmetric	Hungarian	Shapley
Bloom filter [23]	87.24 % (1149)	86.48 % (1139)	87.31 % (1150)
Two-step hash [24]	94.98 % (1251)	94.98 % (1251)	94.98 % (1251)
Tabulation min-hash [37]	49.96 % (658)	51.17 % (674)	54.29 % (715)
Proposed scheme	1.36 % (18)	1.06 % (14)	1.36 % (18)

Referring to the re-identification attack results on the Titanic dataset, detailed in the attached table, our proposed scheme has significantly lowered the re-identification rate to 1.36% for the Symmetric and Shapley matching algorithms, and to 1.06% for the Hungarian algorithm. This is in stark contrast to the Bloom filter and Two-step hash methods, which show re-identification rates of 87.24% and 94.98% respectively across different algorithms.

Our scheme's performance advancement is twofold: First, it ensures a substantial reduction in re-identification rates, thus elevating privacy protection. Second, the computational efficiency is enhanced by our method's less complex and more direct encoding process. This streamlining of processes translates into faster execution times and lower resource consumption without sacrificing the accuracy or integrity of the record linkage.

The improvements are particularly significant given the complexity of the graph-based re-identification attacks, which leverage pattern recognition and relationship inference among the records. Our method's robust resistance to such attacks is evidenced by the decrease in successful re-identification attempts, which ultimately underscores the enhanced privacy and security offered by our scheme.

In essence, our encoding scheme not only strengthens privacy against sophisticated re-identification tactics but also demonstrates a notable leap in performance efficiency.

[Comment 2-8]
In Eq. (3), how the value of n is decided?

(Response 2-8)
We appreciate the reviewers' valuable comments.

In our scheme, *n* is computed using the combination formula $n = \frac{k!}{(k-s)!s!}$, where *k* is the total number of available quasi-identifiers, and *s* is the number of quasi-identifiers selected for encoding. This formula calculates the number of possible unique attribute combinations that can be formed, which directly informs the strength of the anonymization process.

The calculated value of *n* reflects the diversity of the encoded records that our method can generate. A higher *n* implies a greater number of potential encodings, which enhances the difficulty for adversaries attempting re-identification attacks due to increased uncertainty. Hence, *n* is not merely a numerical outcome but a crucial factor that quantifies the level of privacy protection afforded by our encoding method.

[Comment 2-9]
Search stage is completely unclear.

(Response 2-9)
We appreciate the reviewers' valuable comments.

Our manuscript primarily focuses on demonstrating the resilience of our proposed encoding method against re-identification attacks. Consequently, we have not delineated the specifics of the search stage involved in such attacks. For a comprehensive understanding of the search procedure within the context of re-identification attacks, we defer to the foundational work "A Graph Matching Attack on Privacy-Preserving Record Linkage" presented by Vidanage et al. Their paper provides an in-depth exploration of the attack simulation model we employed, detailing the precise mechanisms and stages of the re-identification process, including the search phase.

For readers interested in the technical intricacies of the search strategies and the mechanics of how these attacks are executed, we recommend consulting Vidanage et al.'s publication, which serves as a methodological reference for our simulations.

We believe their work complements ours by elucidating aspects that fall outside the scope of our study's defensive emphasis. Our decision not to elaborate on these procedures in our paper was made to maintain a clear and focused discourse on our encoding method's defensive capabilities. We trust that this approach will facilitate a deeper understanding of our contributions to enhancing privacy preservation within PPRL systems.

[Comment 2-10]
Which entities are involved for key management?

(Response 2-10)
We appreciate the reviewers' valuable comments.

In response to Comment 2-10 regarding key management, we wish to clarify the context and definition of 'key' as it applies to our work, given its potential ambiguity:

1. If 'key' is interpreted in the conventional cryptographic sense, it is important to note that our PPRL (Privacy-Preserving Record Linkage) approach does not employ key-based encryption typically used for user authentication or data encryption. Therefore, in this context, there are no entities involved in key management because key-based encryption is not utilized in our methodology.

2. If 'key' refers to parameters used for encoding in our PPRL framework, these are managed individually by the users performing the Record Linkage (RL) process. Each participating entity in the record linkage process controls its parameters, which act as 'keys' in this sense, to ensure the privacy-preserving aspect of the approach. These parameters, or 'keys', are not shared among entities, aligning with the privacy-preserving goals of our project.

We hope this clarification addresses the concerns regarding key management within our PPRL framework.

[Comment 2-11]
How Eq. (5) improves the performance of the model?

(Response 2-11)
We appreciate the reviewers' valuable comments.

In response to the reviewer's inquiry about the role of Equation (5) in enhancing the model's performance, as discussed in Section 4.3 of our manuscript, we wish to clarify its intended application in our study. Misrepresentation may have led to the perception of this equation as directly aimed at performance improvement within our proposed encoding methodology. We acknowledge this oversight and have amended the presentation to reflect its purpose accurately.

Entropy and information gain serve as metrics to evaluate the security performance of our proposed encoding methods. The related expressions for entropy and information gain are utilized to quantify the amount of information contained within the data, which is crucial for assessing the effectiveness of data anonymization strategies in upholding confidentiality and integrity.

Applying these theoretical constructs in the security realm facilitates a thorough evaluation of the security features of various data anonymization methodologies. Inspired by this established approach, our manuscript employs these principles to appraise the security performance of our proposed technique critically.

The details of the revisions are as follows:

(Revision 2-11)

<table>
<tr><th>Before</th><th>After</th></tr>
<tr><td>

[Section 4.3.1, p.9-10]

Strong Privacy-Preservation

The privacy-preserving performance of the proposed PPRL system was analyzed using entropy. From the perspective of privacy protection, entropy serves as a measure of the degree of randomness or uncertainty in a dataset [36–38]. Entropy essentially signifies the complexity of the anonymization applied to the data, indicating the probability of the original data being inferred by a re-identification attack. A dataset with high entropy exhibits a high degree of randomness. This implies that each record appears with a similar probability or little overlap between records, making it challenging for an attacker to identify a specific entity precisely. Conversely, a dataset with a low entropy displays less randomness and is consequently more predictable, rendering it susceptible to re-identification attacks.

Using entropy, we can calculate the information gain (IG), which is a measure of how much more information an attacker obtains than the original information (information initially possessed or the state of having no information). In other words, we can numerically express using IG how devastating a specific attribute used in the encoding process is if it is leaked and used in a re-identification attack. Hence, we employed

entropy and IG to quantitatively analyze the privacy-preserving performance of the proposed zero-relation encoding method. The entropy of a discrete random variable X, which represents the encoded dataset, is defined in Eq 4. A lower entropy of variable X implies higher certainty, thus a higher probability of predicting the value of X.

$$H(X) = -\sum_{x \in X} p(x) log_2 p(x) \quad (4)$$

Given the value of variable X, we can define the conditional entropy of the discrete random variable Y, which represents the selected attributes, as $H(Y|X)$. $H(Y|X)$ quantifies the uncertainty in predicting the value of Y given variable X (Eq ??). IG measures the uncertainty of the information when inferring the source (Y) using information from the encoded record (X). The IG between the discrete random variables X and Y is defined in Eq 5. A lower IG value implies a greater difficulty in inferring the source from the encoded record, suggesting that the information contained in the variable (Y) does not significantly influence the re-identification of the encoded data.

$$IG(Y|X) = H(Y) - H(Y|X) \quad (5)$$

Table 3 presents the entropy and IG results calculated using the traditional encoding methods and the proposed zero-relation encoding method for PPRL. The bloom filter was excluded because it does not store any information regarding the order or frequency of the elements, making entropy and IG calculations impossible. The dataset encoded using the proposed method exhibited the highest entropy of 17.77, underscoring its capability to anonymize a dataset with randomness and effectively resist re-identification attacks. The IG values for the name, surname, and street address non-unique user attributes selected based on data frequency were calculated in the NCVR dataset. We observed that the more frequent an attribute is, the higher the IG value. This implies that if the street address with the highest IG value is used in the encoding process and subsequently leaked to an attacker, it would have the highest probability of being re-identified. Therefore, researchers or developers using an encoding method based on record attributes must consider the trade-off between the IG value of the attribute and the risk of unique information leakage.

</td><td>

[Section 4.3.1, p.10-11]

4.3.1. Strong Privacy-Preservation

The privacy-preserving efficacy of our proposed PPRL system is substantiated through an analysis utilizing entropy, a measure reflective of randomness or uncertainty within a dataset. Entropy signifies the complexity of the anonymization applied to the data, indicating the probability of the original data being inferred by a re-identification attack [49,50]. High entropy means a complex anonymization process, significantly reducing the likelihood of original data inference through re-identification attacks. This complexity confounds direct attacks and hinders advanced graph-based attacks by obfuscating the relationships within the data structure.

The entropy of a dataset is mathematically represented as:

$$H(X) = -\sum_{x \in X} p(x) \log_2 p(x),$$

where $H(X)$ denotes the entropy of a discrete random variable X, which encapsulates the encoded dataset, and $p(x)$ represents the probability of occurrence of an element x in X.

We extend this notion to calculate the information gain (IG), a metric quantifying the additional information an attacker gains post-attack. This is particularly relevant in assessing the vulnerability of specific attributes within the encoding process:

$$IG(Y|X) = H(Y) - H(Y|X),$$

with $H(Y|X)$ being the conditional entropy of Y given X. A lower IG indicates a more resilient encoding scheme, implying that the attacker's knowledge does not significantly increase, even if certain attributes are compromised.

Our security analysis, as presented in Table 3, juxtaposes the entropy and IG values calculated for both traditional encoding methods and our proposed zero-relationship encoding scheme. Notably, the proposed method achieved a high entropy of 17.77, suggesting an enhanced level of data protection against re-identification.

To confront traditional methods' limitations, we highlight the superior entropy of our method, affirming its capacity to maintain data utility while providing strong privacy. This is contrasted with traditional approaches, which often manifest lower entropy, potentially compromising privacy.

In practical scenarios, such as health data exchange or customer data protection, the proposed method's high entropy and low IG values signal a significant advancement in mitigating the risks of sensitive data exposure. The method is especially pertinent for datasets with inherent attribute correlations, a common vulnerability in PPRL. Our careful selection and encoding of attributes reduce this risk, a crucial factor supported by our findings that higher frequency attributes yield greater IG values.

Conclusively, this rigorous quantitative analysis underscores the zero-relationship encoding method's robustness in theoretical metrics and practical resilience against graph-based re-identification attacks, cementing its role as a pivotal advancement in PPRL privacy measures.

</td></tr>
</table>

[Comment 2-12]

The security analysis is not convincing at all.

(Response 2-12)

We appreciate the reviewers' valuable comments.

In revising the security analysis section, we have aimed to transparently illustrate the foundational principles underpinning our encoding method and its resilience to graph-based re-identification attacks. The revised section now includes a more detailed discussion of the encoding process, emphasizing how it eliminates any direct linkage between the source and the encoded data. This, in turn, substantially diminishes the risk of re-identification, even in the face of sophisticated graph-based attacks.

We are confident that these modifications adequately address your concerns and substantiate the claims made regarding the robustness of our proposed encoding scheme in safeguarding user privacy against re-identification threats.

The details of the revisions are as follows:

(Revision 2-12)

**Before**	**After**
[Section 4, p.9-12]<br>**4.3. Security Analysis**<br>In this section, we present a security analysis to validate the robustness and reliability of the proposed encoding method. The information-hiding ability of the proposed method and its defense performance against the graph-based re-identification attack model were analyzed. Furthermore, we determined whether the proposed encoding method, when applied to the PPRL system, could effectively address the standard threat model scenarios considered by the system.<br>**4.3.1 Strong Privacy-Preservation**<br>The privacy-preserving performance of the proposed PPRL system was analyzed using entropy. From the perspective of privacy protection, entropy serves as a measure of the degree of randomness or uncertainty in a dataset [37–39]. Entropy essentially signifies the complexity of the anonymization applied to the data, indicating the probability of the original data being inferred by a re-identification attack. A dataset with high entropy exhibits a high degree of randomness. This implies that each record appears with a similar probability or little overlap between records, making it challenging for an attacker to identify a specific entity precisely. Conversely, a dataset with a low entropy displays less randomness and is consequently more predictable, rendering it susceptible to re-identification attacks.<br>Using entropy, we can calculate the information gain (IG), which is a measure of how much more information an attacker obtains than the original information (information initially possessed or the state of having no information). In other words, we can numerically express using IG how devastating a specific attribute used in the encoding process is if it is leaked and used in a re-identification attack. Hence, we employed entropy and IG to quantitatively analyze the privacy-preserving performance of the proposed zero-relation encoding method. The entropy of a discrete random variable X, which represents the encoded dataset, is defined in Eq . A lower entropy of variable X implies higher certainty, thus a higher probability of predicting the value of X.<br>$$H(X) = -\sum_{x \in X} p(x) log_2 p(x)$$<br>Given the value of variable X, we can define the conditional entropy of the discrete random variable Y, which represents the selected attributes, as $H(Y\|X)$. $H(Y\|X)$ quantifies the uncertainty in predicting the value of Y given variable X (Eq **??**). IG measures the uncertainty of the information when inferring the source (Y) using information from the encoded record (X). The IG between the discrete random variables X and Y is defined in Eq . A lower IG value implies a greater difficulty in inferring the source from the encoded record, suggesting that the information contained	***[Section 4, p.10-13]***<br>**4.3. Security Analysis**<br>This section addresses the robustness and reliability of our proposed zero-relationship encoding method in preserving user privacy against graph-based re-identification attacks within the context of PPRL systems. Our analysis focuses on the method's capacity to obfuscate information and its defensive efficacy against potential threats.<br>**4.3.1. Strong Privacy-Preservation**<br>The privacy-preserving efficacy of our proposed PPRL system is substantiated through an analysis utilizing entropy, a measure reflective of randomness or uncertainty within a dataset. Entropy signifies the complexity of the anonymization applied to the data, indicating the probability of the original data being inferred by a re-identification attack [49,50]. High entropy means a complex anonymization process, significantly reducing the likelihood of original data inference through re-identification attacks. This complexity confounds direct attacks and hinders advanced graph-based attacks by obfuscating the relationships within the data structure.<br>The entropy of a dataset is mathematically represented as:<br>$$H(X) = -\sum_{x \in X} p(x) \log_2 p(x),$$<br>where $H(X)$ denotes the entropy of a discrete random variable X, which encapsulates the encoded dataset, and $p(x)$ represents the probability of occurrence of an element x in X.<br>We extend this notion to calculate the information gain (IG), a metric quantifying the additional information an attacker gains post-attack. This is particularly relevant in assessing the vulnerability of specific attributes within the encoding process:

Table 2. Information Gain

Encoding method	Entropy	Match Algorithms		
		Symmetric	Hungarian	Shapley
Two-step hash [20]	3.77	0.283	0.347	0.376
Tabulation min-hash [30]	9.965	0.283	0.347	0.376
Proposed	17.77	0.283	0.347	0.376

in the variable (Y) does not significantly influence the re-identification of the encoded data.

$$IG(Y|X) = H(Y) - H(Y|X)$$

Table 2 presents the entropy and IG results calculated using the traditional encoding methods and the proposed zero-relation encoding method for PPRL. The bloom filter was excluded because it does not store any information regarding the order or frequency of the elements, making entropy and IG calculations impossible. The dataset encoded using the proposed method exhibited the highest entropy of 17.77, underscoring its capability to anonymize a dataset with randomness and effectively resist re-identification attacks. The IG values for the name, surname, and street address non-unique user attributes selected based on data frequency were calculated in the NCVR dataset. We observed that the more frequent an attribute is, the higher the IG value. This implies that if the street address with the highest IG value is used in the encoding process and subsequently leaked to an attacker, it would have the highest probability of being re-identified. Therefore, researchers or developers using an encoding method based on record attributes must consider the trade-off between the IG value of the attribute and the risk of unique information leakage.

4.3.2 Defense Against Graph-Based Re-Identification Attack

The graphical representations of encoded datasets are pivotal metrics for assessing the resistance of encoding methods to graph-based re-identification attacks. In the graphs, nodes correspond to a database's set of unique values, while edges indicate the similarities between these values. A high density of nodes means non-unique or similar values, making re-identification based solely on nodes more challenging. However, the presence of numerous edges can aid in identifying similarities between nodes, which can be exploited in re-identification attacks. Contrarily, dispersed nodes could also be problematic, as unique features may facilitate more straightforward identification. Ideally, an effective encoding method would produce a graph with fewer nodes and edges that are neither densely packed nor dispersed. The balanced distribution of graphs makes both feature extraction and re-identification more difficult. Using the Titanic and NCVR datasets, encoding was conducted using four techniques: bloom filter, two-step hash, table min-hash, and the proposed zero-relationship encoding method. Subsequently, the encoded datasets were transformed into graphical representations to assess their resistance to potential attacks visually. Fig 6 shows the characteristics of each encoding method when the encoded data are graphically represented.

- Bloom Filter and Two-Step Hash: For both the Titanic and NCVR datasets, these methods resulted in graphs with densely interconnected nodes, indicating that they possess inherent vulnerabilities to re-identification attacks.
- Tabulation Min-hash: The graph appears slightly less dense for the Titanic dataset than the graphs of the two previously discussed encoding methods. However, it still offers many extractable features, making it vulnerable to re-identification attacks. For the more complex NCVR dataset, the graph illustrates a densely packed node graph with many edges akin to those produced by the other two methods. This shows that the tabulation min-hash remains susceptible to graph-based re-identification attacks.
- Zero-Relationship Encoding: Our proposed method showcased exceptional resistance against re-identification. In the case of the Titanic dataset, the graph reveals nodes that are not densely packed but are uniformly spread with few edges. In contrast to the Titanic dataset, for the NCVR dataset, which inherently has a structured appearance owing to its multiple attributes, the graph still exhibited fewer edges and nodes clustered in multiple groups. This distribution makes feature extraction more challenging than the graphs derived from traditional encoding methods.

The graph representations show the robust defensive performance of the zero-relationship encoding method against graph-based re-identification attacks, particularly compared with other encoding methods.

Table 3. Information Gain

Encoding method	Entropy	Match Algorithms		
		Symmetric	Hungarian	Shapley
Two-step hash [24]	3.77	0.283	0.347	0.376
Tabulation min-hash [37]	9.965	0.283	0.347	0.376
Proposed	17.77	0.283	0.347	0.376

$$IG(Y|X) = H(Y) - H(Y|X),$$

with $H(Y|X)$ being the conditional entropy of Y given X. A lower IG indicates a more resilient encoding scheme, implying that the attacker's knowledge does not significantly increase, even if certain attributes are compromised.

Our security analysis, as presented in Table 3, juxtaposes the entropy and IG values calculated for both traditional encoding methods and our proposed zero-relationship encoding scheme. Notably, the proposed method achieved a high entropy of 17.77, suggesting an enhanced level of data protection against re-identification.

To confront traditional methods' limitations, we highlight the superior entropy of our method, affirming its capacity to maintain data utility while providing strong privacy. This is contrasted with traditional approaches, which often manifest lower entropy, potentially compromising privacy.

In practical scenarios, such as health data exchange or customer data protection, the proposed method's high entropy and low IG values signal a significant advancement in mitigating the risks of sensitive data exposure. The method is especially pertinent for datasets with inherent attribute correlations, a common vulnerability in PPRL. Our careful selection and encoding of attributes reduce this risk, a crucial factor supported by our findings that higher frequency attributes yield greater IG values.

Conclusively, this rigorous quantitative analysis underscores the zero-relationship encoding method's robustness in theoretical metrics and practical resilience against graph-based re-identification attacks, cementing its role as a pivotal advancement in PPRL privacy measures.

4.3.2. Defense Against Graph-Based Re-Identification Attack

Graphical representations of encoded datasets serve as critical indicators of an encoding method's resilience to graph-based re-identification attacks. In these representations, nodes represent unique values from a database, and edges denote similarities among these values. A graph's resilience to re-identification attacks is inversely proportional to the density of its nodes and edges: denser graphs, with many interconnected nodes, make them vulnerable to attacks as they facilitate the extraction of similarities that can lead to re-identification. Conversely, dispersed nodes could pose challenges, as unique features in such a distribution may facilitate easier identification of individual records. Ideally, an effective encoding method would produce a graph with fewer nodes and edges that are neither densely packed nor dispersed.

In our analysis, we employed the Titanic and NCVR datasets to evaluate the resistance of various encoding techniques—including bloom filter, two-step hash, table min-hash, and our encoding method—against graph-based re-identification attacks. The encoded datasets were visually transformed into graphs, allowing for an assessment of each method's defensive capabilities. Fig 6 shows the characteristics of each encoding method when the encoded data are graphically represented.

- **Bloom Filter and Two-Step Hash:** These methods produced densely interconnected graphs for both the Titanic and NCVR datasets, revealing a

fundamental susceptibility to re-identification attacks. The dense node connectivity in these graphs implies a higher risk of attribute similarity exploitation, which can lead to successful re-identification.

- **Tabulation Min-hash:** The graph for the Titanic dataset appeared slightly less dense compared to the previous methods, yet it still exhibited numerous extractable features that render it vulnerable to attacks. The NCVR dataset's graph, characterized by densely packed nodes and numerous edges, further confirmed the tabulation min-hash method's vulnerability to re-identification.
- **Zero-Relationship Encoding:** The zero-relationship encoding method demonstrated superior resistance to re-identification attacks. The Titanic dataset's graph, characterized by uniformly spread nodes with minimal edges, and the NCVR dataset's graph, with its nodes clustered into multiple groups and fewer edges, illustrate the method's effectiveness. The reduced node and edge density significantly complicates feature extraction and subsequent re-identification attempts, highlighting the zero-relationship encoding method's robustness.

In summary, the comparative analysis of graphical representations underscores the zero-relationship encoding method's enhanced defense against graph-based re-identification attacks. By minimizing node density and edge connectivity, our method substantially reduces the feasibility of exploiting attribute similarities, thereby preserving user privacy more effectively within PPRL frameworks.

4.3.3. Threat Model Scenario

PPRL systems facilitate the exchange of anonymized information among entities while safeguarding against unauthorized data breaches and re-identification threats. These systems, operating within a networked environment, are inherently exposed to a spectrum of security vulnerabilities, ranging from passive eavesdropping to active interference such as spoofing, sniffing, and snooping. The implications of such vulnerabilities extend to the unauthorized decryption and misuse of sensitive personal information, necessitating a robust defense mechanism inherent to the PPRL framework.

The design of the PPRL system is predicated on a semi-trust model among participating entities, who, while adhering to the established record linkage protocols, could potentially harbor honest-but-curious or outright malicious intents. The collaborative nature of the PPRL process involves the sharing of information necessary for record linkage, introducing a potential vector for the exploitation of anonymized data, thereby elevating the risk of re-identification without explicit consent from the data subjects.

Addressing the spectrum of threats, from honest-but-curious participants to external adversaries with malicious intent, the proposed zero-relationship encoding method employs a strategic limitation on the utilization of record attributes during the linkage process. This method leverages a selective subset of quasi-identifiers—attributes sufficiently generic yet vital for the linkage process such as name, sex, and age—thereby ensuring that the exposure of any single attribute does not compromise the integrity of the entire dataset. Moreover, the encoding process is further secured through the confidential agreement among participants on key parameters such as the hash function list and the attribute selection criteria, thereby obfuscating the encoding logic from potential adversaries.

The zero-relationship encoding approach fundamentally disrupts the direct correlation between the source and encoded records, thereby mitigating the risk associated with conventional encoding methodologies that preserve certain relational aspects susceptible to exploitation. By amalgamating multiple attributes to generate encoded values devoid of direct linkage to the original data, the method significantly impedes the adversaries' ability to apply cryptographic or pattern analysis techniques for data re-identification. This encoding paradigm, by obfuscating the relational mappings, effectively nullifies the utility of graph-based analysis tools in deciphering the encoded datasets, thereby fortifying the privacy assurances provided by the PPRL system.

In summary, the zero-relationship encoding method presents a comprehensive defense framework against the multifaceted threat landscape associated with PPRL systems. By ingeniously dissociating the encoded data from its source through a blend of attribute selection rigor and encoding complexity, the method not only thwarts attempts at unauthorized re-identification but also establishes a new benchmark for privacy preservation within the domain of PPRL.

[Comment 2-13]
What is the source of the dataset? Whether it is authentic or not? Mention clearly.

(Response 2-13)
We appreciate the reviewers' valuable comments.

In response to Comment 2-13 regarding the source of the dataset, we have made the necessary changes from footnotes to reference format, as per the journal's requirements, to clearly cite the origin of the datasets used in our experiments.

Specifically, the dataset utilized, known as the Titanic and North Carolina Voter Records (NCVR) dataset, is a widely recognized open dataset accessible to the public. It is a legitimate

source of data commonly used in PPRL (Privacy-Preserving Record Linkage) and other data science research to benchmark and validate methodologies due to its comprehensive and illustrative nature. We have now included this information in the references section to provide clear documentation and confirmation of the datasets' authenticity and common usage in the field.

We hope this modification and clarification meet the journal's standards and address the concerns regarding the datasets' source and authenticity.

The details of the revisions are as follows:

(Revision 2-13)

**Before**	**After**
[-]	***[Reference, p.15]***<br>47. Frank E Harrell Jr TC. Titanic dataset; 2017. Available from: https://www.openml.org/d/40945.<br><br>48. Christen P. Preparation of a real temporal voter data set for record linkage and duplicate detection research. Te chnical Rep ort The Australian National University. 2014;.

[Comment 2-14]
How the results of Figure 3 are generated?

(Response 2-14)
We appreciate the reviewers' valuable comments.

The Figure 3 presented in the paper, titled "Re-identification rates in the Titanic dataset: Comparing encoding methods and matching algorithms," was created using the results from experiments utilizing a graph-based re-identification attack simulator provided by *Vidanage et al.,*[1] along with the Titanic dataset. The simulations compared the proposed method with prominent encoding methods such as Bloom filter, two-step hash, and tabulation min-hash encodings, aiming to enhance the security of Privacy-Preserving Record Linkage (PPRL). The proposed method demonstrated the lowest re-identification rates across all matching algorithms.

To aid in the interpretation of simulation results, a table displaying the re-identification rates was added to the existing figure. All graphical representations of simulation results were gen-

[1] Vidanage A, Christen P, Ranbaduge T, Schnell R. A graph matching attack on privacy-preserving record linkage. Proceedings of the 29th ACM International Conference on Information & Knowledge Management. 2020; p. 1485–1494

erated using the matplotlib.pyplot library within the Python 3 environment of Jupyter Notebook platform. This ensured accurate and clear visualization of the data obtained from the experiments. The simulation result graphs and re-identified records were provided to precisely convey the outcomes of the research.

The details of the revisions are as follows:

(Revision 2-14)

<table>
<tr><th>Before</th><th>After</th></tr>
<tr><td>

[-]

</td><td>

[Section 1, p.2]

Figure 3. Re-identification rates in the Titanic dataset: Comparing encoding methods and matching algorithms.

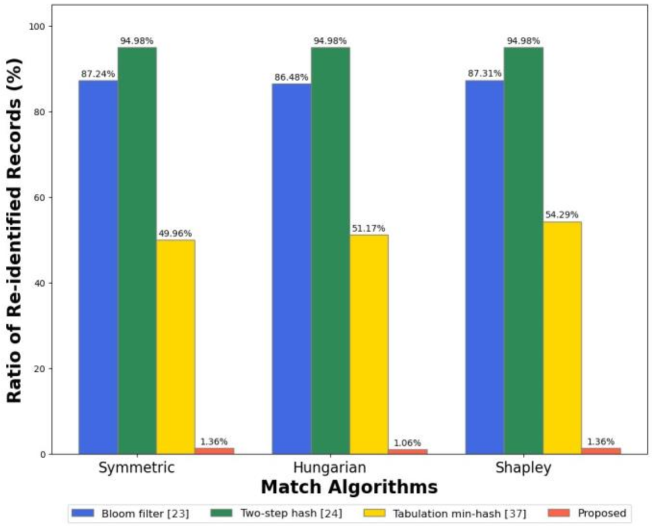

<table>
<tr><th rowspan="2">Encoding method</th><th colspan="3">Match Algorithms</th></tr>
<tr><th>Symmetric</th><th>Hungarian</th><th>Shapley</th></tr>
<tr><td>Bloom filter [23]</td><td>87.24 % (1149)</td><td>86.48 % (1139)</td><td>87.31 % (1150)</td></tr>
<tr><td>Two-step hash [24]</td><td>94.98 % (1251)</td><td>94.98 % (1251)</td><td>94.98 % (1251)</td></tr>
<tr><td>Tabulation min-hash [37]</td><td>49.96 % (658)</td><td>51.17 % (674)</td><td>54.29 % (715)</td></tr>
<tr><td>Proposed scheme</td><td>1.36 % (18)</td><td>1.06 % (14)</td><td>1.36 % (18)</td></tr>
</table>

</td></tr>
</table>

[Comment 2-15]

Technical details about results are missing.

(Response 2-15)

We appreciate the reviewers' valuable comments.

We have carefully considered the feedback regarding our results' lack of technical details. In this revision, we present a more detailed analysis, including specific re-identification rates for each encoding method and matching algorithm, along with a discussion of the statistical measures used to evaluate the significance of our findings.

The details of the revisions are as follows:

(Revision 2-15)

Before	After
[Section 4, p.7-8] **Results: Evaluation and Discussion** <br>This section evaluates the privacy-preserving performance and feasibility of the proposed zero-relationship encoding method by simulating the re-identification attack outlined in [18]. <br>4330, and 4328 records, indicating that most of the records were re-identified. The tabulation min-hash showed improved performance with 1786, 1768, and 1802 re-identified records. However, the proposed zero-relationship method demonstrated complete resistance to re-identification, with zero re-identified records for all matching algorithms. These results indicate that the zero-relationship encoding method offers excellent resistance to re-identification attacks by minimizing the risk of re-identification. Simulation results confirmed that the durability of the zero-relationship encoding method against re-identification attacks increased as the number of attributes used in the encoding process increased. We observed a lower re-identification rate in the experimental results of the NCVR dataset, which used three attributes, compared to the Titanic dataset, which used two attributes. Building on this finding, we designed experiments to quantitatively assess the impact of attribute count on performance metrics such as encoding time, feature extraction time, and re-identification attack time. Fig **??** shows the durability of the zero-relationship encoding method against re-identification attacks depending on the number of attributes. We verified that the increase in the number of attributes used in the encoding process increases the time spent on encoding. The time required for feature extraction and re-identification attacks also increased. Particularly when more than 14 attributes were used with encoding, we observed a sharp increase in the time required. This suggests that using numerous attributes for encoding results in the creation of a form without specific meaning, making feature extraction and re-identification more challenging. Although an increased number of records used in the encoding process requires additional computation time, the simulation results confirmed that it can demonstrate strong resilience against re-identification attacks. Our approach of minimizing the relationship between source and encoded records to preserve privacy experimentally proved to be effective in defending against re-identification attacks.	***[Section 4, p.9-10]*** **4. Results: Evaluation and Discussion** <br>**4.1. Simulation Setup** <br>The performance of the proposed encoding method was assessed through experiments utilizing a re-identification attack simulator provided by [22]. The simulator, implemented in Python 2.7, was executed on a PC with a 64-bit Intel(R) Xeon(R) W-3245 3.20 GHz CPU and 256 GB of memory, running Ubuntu 20.04.1. Access to the utilized simulator is available via the link provided by the authors at https://dmm.anu.edu.au/pprlattack/. For the simulations, two types of public datasets were employed: the Titanic dataset [47] and the North Carolina Voter Registration (NCVR) database [48]. The Titanic dataset utilized attributes such as the first and last names of 1,317 passengers. The NCVR dataset (accessed in June 2014) contains information on 224,073 North Carolina voters across 19 attribute fields. Due to numerous null values across fields, which could introduce noise or errors if used without preprocessing, a subset of 4,401 voter records without any null values was employed for accurate simulation. In the simulations based on the NCVR dataset, attributes with the highest and lowest cardinality values were excluded, and a subset of the remaining 17 attributes was randomly selected for use. <br>**4.2. Performance Evaluation** <br>This section evaluates the proposed zero-relationship encoding method through a graph-based re-identification attack simulator. Our assessment focused on the method's effectiveness in preserving privacy by measuring the ratio of re-identified records. The encoding methods included in our comparison are the Bloom filter [36], Two-step hash [24], and Tabulation min-hash [37], all representing established privacy preservation techniques. The experiments utilized two distinct datasets to conduct the evaluations: the Titanic and NCVR datasets, the results of which are illustrated in Figures 3 and 4, respectively. We observed that the zero-relationship encoding method significantly reduced the ratio of re-identified records across all matching algorithms compared to other encoding methods. The simulation results demonstrate that our encoding method effectively counters re-identification attacks by severing the explicit relationship between records. The experiments utilizing the Titanic dataset demonstrated that traditional encoding methods like the Bloom filter, Two-step hash, and Tabulation min-hash resulted in a significant proportion of re-identified records. On average, these methods experienced re-identification rates of approximately 87%, 94.98%, and 50%, respectively, across various matching algorithms. In stark contrast, the proposed zero-relationship encoding method exhibited a remarkably lower average re-identification rate of around 1.3%, underscoring its superior performance in preserving privacy. The NCVR dataset provided further evidence of the proposed method's robustness. The Bloom filter re-identified over 3,990 records for each matching algorithm, while the Two-step hash method showed a near-complete re-identification rate. The Tabulation min-hash offered an improved outcome yet allowed more than 1700 records to be re-identified. Strikingly, the proposed method achieved a zero re-identification rate, demonstrating an outstanding defense against re-identification attacks. The proposed zero-relationship encoding method eliminates any linkage between the source and the encoded records, thwarting re-identification attempts. It employs a novel anonymization process that leverages the attributes of the source record to produce an outcome with minimal ties to the original data, thus significantly enhancing privacy preservation. Our method utilizes cardinality to prioritize the security of attributes. We <br>exclude those with extremely high or low values to balance utility and security. The min-hash function is employed to obtain the smallest hash value for selected attributes, combined using XOR operations. This process not only enhances the protection of the dataset by transforming attribute data but also denies adversaries by obscuring the original information. This approach introduces uncertainty in identifying and classifying anonymized entities, significantly impairing the efficacy of potential attacks. Our study further examined the relationship between the number of attributes used in the encoding process and the performance of the proposed method. Fig 5 shows the results of a performance measurement experiment of the unrelated encoding method depending on the number of attributes used for encoding. The NCVR dataset, which used three attributes, exhibited a lower re-identification rate than the Titanic dataset with two attributes. The graph demonstrates that as the number of attributes increases, there is a notable impact on both encoding and feature generation times. Specifically, a substantial rise in time required is observed as we move from 9 to 14 attributes. Despite this increase in computational effort, the associated rise in re-identification time indicates an enhancement in privacy protection. This trade-off highlights the method's scalability and potential to bolster privacy defenses with increased attribute counts, as higher complexity leads to reduced re-identification success. This nuanced understanding of performance dynamics is crucial for implementing the method in practical scenarios where attribute quantity and processing capabilities must be balanced. The experiments conducted underscore the effectiveness of the proposed zero-relationship encoding method in mitigating the risk of data re-identification. Minimizing the relationship between the source and the encoded records provides a promising approach to privacy preservation in PPRL. <br>Figure 3. Re-identification rates in the Titanic dataset: Comparing encoding methods and matching algorithms.

Simulation Setup

This study simulates a scenario in which an attacker attempts to re-identify anonymized records using a plain data set. All simulations were performed on Ubuntu 20.04.1 with an Intel(R) Xeon(R) W-3245 3.20 GHz CPU and 256 GB of memory. Two datasets were used in the security durability evaluation simulation against re-identification attacks: the Titanic[1] and North Carolina Voter Registration (NCVR) databases[2]. In the Titanic dataset, we used attributes such as the first and last names of 1,317 passengers for encoding. The NCVR dataset, which was created in June 2014, was used in the simulation as both encoding and plain data for re-identification. This dataset includes information on 224,073 voters from North Carolina and comprises 19 attribute fields. However, the NCVR dataset contains numerous null values. Using these null values in the anonymization process can introduce noise or errors into the results. Therefore, to ensure an accurate simulation, we used information from 4,401 voters that did not contain any null values. In the simulation using the NCVR dataset, we randomly selected attributes for encoding from a pool of 17, excluding the two attributes with the highest and lowest cardinality values from a total of 19 user attributes.

Performance Evaluation

To evaluate the security performance of the proposed method, we measure the ratio of re-identified records. We also compare the ratio of re-identified records of the proposed scheme with the existing encoding methods, such as Bloom filter [19], Two-step hash [20], and Tabulation min-hash [30]. The results of the re-identification attack simulations for each dataset are presented in Fig **??** and Table 2. The primary metric of the evaluation, the ratio of re-identified records, was markedly lower with the zero-relationship method than with the other three encoding algorithms.

The simulation results using the Titanic dataset shown in Fig **??** confirmed the different security performances depending on the encoding algorithm. In Fig **??**, the x-axis represents the matching algorithms (symmetric, Hungarian, Shapley) used to compare the similarity between the plain data and the data encoded by each algorithm. The y-axis indicates the ratio of re-identified records. Using the bloom filter, 1149, 1139, and 1150 records were re-identified for the Symmetric, Hungarian, and Shapley matching algorithms, respectively. In the case of the two-step hash, 1251 records were re-identified across all matching algorithms. The tabulation min-hash resulted in 658, 674, and 715 re-identified records, whereas the proposed zero-relationship method had only 18, 14, and 18 re-identified records. A similar trend was observed in the simulation results for the NCVR dataset in Table 2. The bloom filter resulted in 3993, 3999, and 3996 re-identified records. For the two-step hash, the re-identification counts were 4327,

[1]https://en.wikipedia.org/wiki/Passengers_of_the_Titanic
[2]http://dl.ncsbe.gov/

Table 2. Re-identification rates in NCVR dataset: Comparing encoding methods and matching algorithms.

Encoding method	Match Algorithms		
	Symmetric	Hungarian	Shapley
Bloom filter [19]	87.24 %	86.48 %	87.31 %
Two-step hash [20]	94.98 %	94.98 %	94.98 %
Tabulation min-hash [30]	49.96 %	51.17 %	54.29 %
Proposed	0 %	0 %	0 %

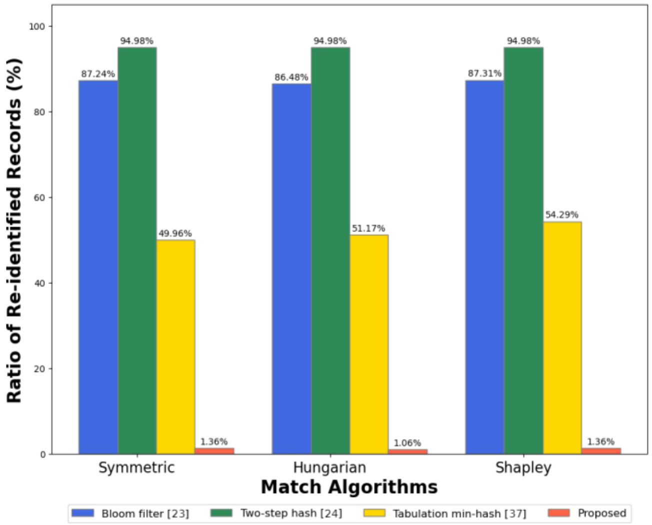

Encoding method	Match Algorithms		
	Symmetric	Hungarian	Shapley
Bloom filter [23]	87.24 % (1149)	86.48 % (1139)	87.31 % (1150)
Two-step hash [24]	94.98 % (1251)	94.98 % (1251)	94.98 % (1251)
Tabulation min-hash [37]	49.96 % (658)	51.17 % (674)	54.29 % (715)
Proposed scheme	1.36 % (18)	1.06 % (14)	1.36 % (18)

Figure 4. Re-identification rates in the NCVR dataset: Comparing encoding methods and matching algorithms.

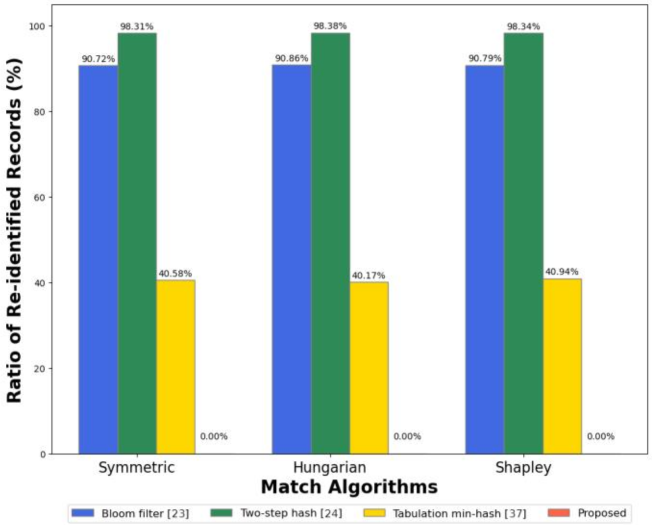

Encoding method	Match Algorithms		
	Symmetric	Hungarian	Shapley
Bloom filter [23]	90.72 % (3993)	90.86 % (3999)	90.79 % (3996)
Two-step hash [24]	98.31 % (4327)	98.38 % (4330)	98.34 % (4328)
Tabulation min-hash [37]	40.58 % (1786)	40.17 % (1768)	40.94 % (1802)
Proposed scheme	0 % (0)	0 % (0)	0 % (0)

Figure 5. Performance of the zero-relationship encoding method according to the number of attributes used for encod-
ing

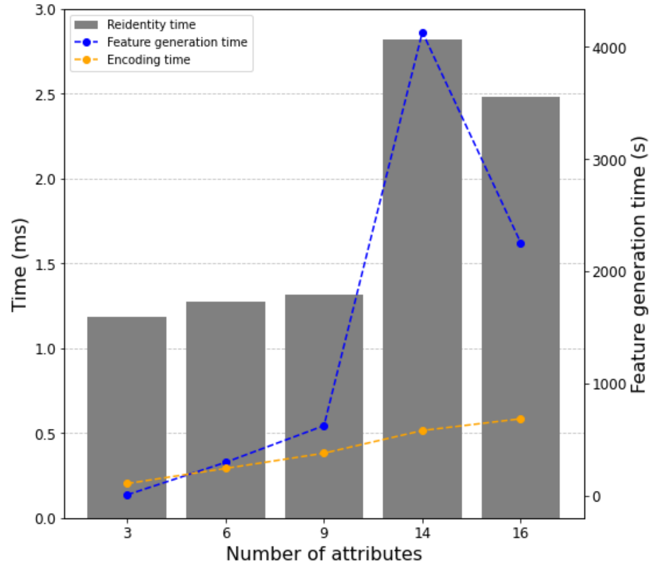

[Comment 2-16]
How the training is done?

(Response 2-16)
We appreciate the reviewers' discerning question regarding the training process

To clarify, our proposed scheme does not employ a machine learning model and, as such, does not entail a training phase. The mention of a decision model within our work refers to a deterministic process that is part of the attack simulation rather than a learnable algorithm requiring training.

The experimental setup utilized in our research is based on the decision model described by Vidanage et al. in their study "A Graph Matching Attack on Privacy-Preserving Record Linkage." This model operates by extracting features from both plaintext and encoded text graphs to perform node-by-node matching. The effectiveness of this model in simulating graph-based re-identification attacks underscores the robustness of our encoding method against such privacy threats.

We hope this clarifies the context in which the term 'decision model' was used and confirms that there is no training process associated with the proposed encoding technique.

[Comment 2-17]
What is the novelty of this work? It is hard to identify from the current version of this paper.

(Response 2-17)
We appreciate the reviewers' valuable comments.

The innovative aspect of our study lies in the development of the Zero-Relationship Encoding Method designed specifically for privacy-preserving record linkage (PPRL). This method strategically eliminates any computable relationship between the original and the encoded

records, thereby addressing the vulnerability of existing methods to graph-based re-identification attacks.

Our contribution is twofold: First, we introduce the concept of 'data relatedness' in PPRL and show through simulations how reducing this relatedness significantly diminishes the risk of re-identification. Second, we propose a new encoding algorithm that applies a distinctive combination of hash functions and XOR operations to anonymize data, which has not been explored in previous studies.

This novel approach not only enhances privacy but also retains the utility of the data for linkage purposes, setting our work apart from existing PPRL techniques. We believe this advancement constitutes a significant contribution to the field of data privacy.

[Comment 2-18]

Key terms of the equations must be defined.

(Response 2-18)

We appreciate the reviewers' valuable comments.

In response to Comment 2-18 regarding the definition of key terms used in the equations, we would like to clarify that all key terms and notations have been defined clearly in Table 1 of our manuscript. We have conducted a thorough review to ensure that there are no omissions and can confirm that each term used within the equations has been accurately and explicitly defined.

We understand the importance of clarity in presenting our research and apologize for any confusion. However, upon reevaluation, we have found that all relevant terms are indeed listed and described in Table 1, as initially provided.

We hope this addresses the concern raised and reaffirms the completeness and clarity of the definitions within our paper.

[Section 3, p.5]

Table 1. Notations

Symbol	Description
DB_p	Database of participant
DB_p^{τ}	Anonymized database
P	Participant in PPRL
$attr$	Attribute of an entity
k	Number of attributes
μ	Hash seed
H	List of hash functions
e_n	Entity
e^{γ}	Entity with selected attributes
ϕ	Min-hash XOR set
e^{τ}	Anonymized entity
$Zero(DB_p, \mu, s)$	Zero-relationship encoding method
$Unique(DB_p)$	Number of unique values in the attributes
$Total(DB_p)$	Total number of values in the attributes
$Card(attr_i)$	Degree of duplication for attributes
δ	Security rank of the attributes
s	Number of attributes for selection
γ	Selected attribute set
$attr_i'$	hash value of the attribute
ζ	Hashed attributes set in γ
θ	Combination of elements in ζ
$\Xi(\cdot)$	Record linkage decision model

[Comment 2-19]

Use a well-known software to draw the diagrams of the results section.

(Response 2-19)
We appreciate the reviewers' valuable comments.

We have crafted all graphical representations of the experimental outcomes using matplotlib.pyplot library within the Python 3 environment, hosted on the Jupyter Notebook platform. This approach ensures precision and clarity when illustrating the data obtained from our simulation exercises. The visual depictions, including those elucidating the encoded records, have been generated with the utmost care to convey our study's intricate details and significant findings accurately. This well-known visualization tool underscores our results with graphical excellence and interpretability standards.

[Comment 2-20]
The organization of the paper must be improved. The paper must be formatted properly.

(Response 2-20)
We appreciate the reviewers' valuable comments.

In response to your comments, we have carefully revised our manuscript following the submission guidelines provided by PLOS ONE. We ensured that our paper's structure, formatting, and overall presentation aligned with the journal's standards, aiming to enhance readability and coherence. To further improve our manuscript, we have examined and taken inspiration from several papers published in PLOS ONE, particularly regarding organization and content structuring. These papers served as benchmarks for refining our approach:

- On the effectiveness of graph matching attacks against privacy-preserving record linkage

- Encoding edge type information in graphlets

- A novel privacy-preserving biometric authentication scheme

- Local differential privacy protection for wearable device data

These adjustments have significantly improved the manuscript's organization and adherence to the PLOS ONE formatting guidelines. We are confident that these changes address your concerns and enhance the paper's quality.

[Comment 2-21]
Improve the English language.

(Response 2-21)
We appreciate the reviewers' valuable comments.

In response to your invaluable feedback regarding enhancing the English language quality within our manuscript, we have undertaken a comprehensive revision process to elevate our text's linguistic precision and eloquence. To this end, we have engaged the services of a professional editing firm specializing in academic English, ensuring that our manuscript undergoes a rigorous language polishing procedure. Enclosed herewith, for your perusal, is the confirmation of service from the editing firm, attesting to the thorough review and refinement undertaken. Additionally, we have meticulously reviewed and amended other aspects of the manuscript to fortify its overall academic rigor and clarity. This endeavor reflects our unwavering commitment to presenting our research with the utmost linguistic integrity and scholarly excellence.

(Revision 2-21)

Before	After
[-]	editage<br><br>Letter from the editor<br><br>Thank you for using Editage services.<br><br>Please review the corrected file content, comments left by the editor for you, and the message below.<br>If you have any questions about the editor's corrections or want to convey responses to the editor's comments, you can submit them through the 'Submit a Question' feature in EditageOnline™ http://app.editage.co.kr/). Since the questions will be sent directly to the editor, please write them in English.<br>Because language errors can occur even in minor revisions, we recommend getting your manuscript reviewed by a native speaker before final submission. If you need additional corrections from the editor for the parts you have revised, you can request a re-review at a 60% discounted rate through the 'Request for Re-review' feature in the Editage online system.<br>Additionally, your feedback on this work will help us provide you with improved services. You can leave feedback for the work through the completed job download window or review section in EditageOnline™.<br>▸ Can Editage be mentioned in the Acknowledgement?<br>Many authors mention Editage in the Acknowledgement after using Editage's editing services. Guidelines provided by prestigious editorial committees like ICMJE specify that companies that have helped with editing or writing should be mentioned in the Acknowledgement. Such acknowledgment reinforces to journal editors/reviewers that the paper's English has been thoroughly reviewed and meets the standards required for publication.<br>If you wish to mention Editage in the Acknowledgement, you can briefly state as follows:<br>We would like to thank Editage (www.editage.co.kr) for English language editing.<br><br>Thank you,<br>Editage Team<br><br>**Quick tip**<br>**Guideline**<br>Wordiness (the use of many words to convey an idea) should be avoided in academic writing.<br><br>**Explanation**<br>The use of too many words to convey one idea can muddle the message and divert the reader's attention. Therefore, ideas need to be conveyed as concisely as possible. One way of doing this is to use a direct verb (action) instead of using a noun and verb. For example, "considerations must be made for" in the sentence below can be simply expressed as "must be considered." Note, however, that this requires slightly restructuring the sentence to facilitate the change.<br><br>**Example**<br>Avoid: In the PPRL system, *considerations must be made for* potential threats from external or internal malicious adversaries.<br><br>Better: In the PPRL system, potential threats from external or internal malicious adversaries *must be considered.*

[Comment 2-22]

The Reference section must be improved significantly.

(Response 2-22)

We appreciate the reviewers' valuable comments.

We have revised the section more abundantly by referring to the papers and additional papers that you recommended the Reference section by reflecting the comments you gave us.

The details of the revisions are as follows:

(Revision 2-22)

<table>
<tr><th>Before</th><th>After</th></tr>
<tr><td>

[Section 7, p.12]

References

1. Fernández-Alemán JL, Señor IC, Lozoya PÁO, Toval A. Security and privacy in electronic health records: A systematic literature review. Journal of biomedical informatics. 2013;46(3):541–562.
2. Abouelmehdi K, Beni-Hessane A, Khaloufi H. Big healthcare data: preserving security and privacy. Journal of big data. 2018;5(1):1–18.
3. Rajpurkar P, Chen E, Banerjee O, Topol EJ. AI in health and medicine. Nature Medicine. 2022;28(1):31–38.
4. Tian S, Yang W, Le Grange JM, Wang P, Huang W, Ye Z. Smart healthcare: making medical care more intelligent. Global Health Journal. 2019;3(3):62–65.
5. Price WN, Cohen IG. Privacy in the age of medical big data. Nature medicine. 2019;25(1):37–43.
6. Hariri RH, Fredericks EM, Bowers KM. Uncertainty in big data analytics: survey, opportunities, and challenges. Journal of Big Data. 2019;6(1):1–16.
7. Ghasemaghaei M, Calic G. Can big data improve firm decision quality? The role of data quality and data diagnosticity. Decision Support Systems. 2019;120:38–49.
8. Nguyen L, Stoové M, Boyle D, Callander D, McManus H, Asselin J, et al. Privacy-preserving record linkage of deidentified records within a public health surveillance system: evaluation study. Journal of Medical Internet Research. 2020;22(6):16757.
9. Bampoulidis A, Markopoulos I, Lupu M. Prioprivacy: a local recoding k-anonymity tool for prioritised quasi-identifiers. IEEE/WIC/ACM International Conference on Web Intelligence-Companion Volume. 2019; p. 314–317.
10. Kaur G, Agrawal S; Springer. Differential privacy framework: Impact of quasi-identifiers on Anonymization. Proceedings of 2nd International Conference on Communication, Computing and Networking. 2019; p. 35–42.
11. Abdelhameed SA, Moussa SM, Khalifa ME. Privacy-preserving tabular data publishing: a comprehensive evaluation from web to cloud. Computers & Security. 2018;72:74–95.
12. Al-Dulaimi A, Mumtaz S, Al-Rubaye S, Zhang S, Chih-Lin I. A framework of network connectivity management in multi-clouds infrastructure. IEEE Wireless Communications. 2019;26(3):104–110.
13. Wu PY, Cheng CW, Kaddi CD, Venugopalan J, Hoffman R, Wang MD. -Omic and electronic health record big data analytics for precision medicine. IEEE Transactions on Biomedical Engineering. 2016;64(2):263–273.
14. Wang Y, Kung L, Wang WYC, Cegielski CG. An integrated big data analytics-enabled transformation model: Application to health care. Information & Management. 2018;55(1):64–79.
15. Jain P, Gyanchandani M, Khare N. Differential privacy: its technological prescriptive using big data. Journal of Big Data. 2018;5(1):1–24.
16. Christen P, Ranbaduge T, Vatsalan D, Schnell R. Precise and fast cryptanalysis for Bloom filter based privacy-preserving record linkage. IEEE Transactions on Knowledge and Data Engineering. 2018;31(11):2164–2177.
17. Heng Y, Armknecht F, Chen Y, Schnell R. On the effectiveness of graph matching attacks against privacy-preserving record linkage. PloS one. 2022;17(9):0267893.
18. Vidanage A, Christen P, Ranbaduge T, Schnell R. A graph matching attack on privacy-preserving record linkage. Proceedings of the 29th ACM International Conference on Information & Knowledge Management. 2020; p. 1485–1494.

</td><td>

[Section 7, p.14]

References

1. Fernández-Alemán JL, Señor IC, Lozoya PÁO, Toval A. Security and privacy in electronic health records: A systematic literature review. Journal of biomedical informatics. 2013;46(3):541–562.
2. Abouelmehdi K, Beni-Hessane A, Khaloufi H. Big healthcare data: preserving security and privacy. Journal of big data. 2018;5(1):1–18.
3. Rajpurkar P, Chen E, Banerjee O, Topol EJ. AI in health and medicine. Nature Medicine. 2022;28(1):31–38.
4. Tian S, Yang W, Le Grange JM, Wang P, Huang W, Ye Z. Smart healthcare: making medical care more intelligent. Global Health Journal. 2019;3(3):62–65.
5. Price WN, Cohen IG. Privacy in the age of medical big data. Nature medicine. 2019;25(1):37–43.
6. Wang M, Yi H, Jiang F, Lin L, Gao M. Review on offloading of vehicle edge computing. Journal of Artificial Intelligence and Technology. 2022;2(4):132–143.
7. Hariri RH, Fredericks EM, Bowers KM. Uncertainty in big data analytics: survey, opportunities, and challenges. Journal of Big Data. 2019;6(1):1–16.
8. Ghasemaghaei M, Calic G. Can big data improve firm decision quality? The role of data quality and data diagnosticity. Decision Support Systems. 2019;120:38–49.
9. Nguyen L, Stoové M, Boyle D, Callander D, McManus H, Asselin J, et al. Privacy-preserving record linkage of deidentified records within a public health surveillance system: evaluation study. Journal of Medical Internet Research. 2020;22(6):16757.
10. Bampoulidis A, Markopoulos I, Lupu M. Prioprivacy: a local recoding k-anonymity tool for prioritised quasi-identifiers. IEEE/WIC/ACM International Conference on Web Intelligence-Companion Volume. 2019; p. 314–317.
11. Kaur G, Agrawal S; Springer. Differential privacy framework: Impact of quasi-identifiers on Anonymization. Proceedings of 2nd International Conference on Communication, Computing and Networking. 2019; p. 35–42.
12. Abdelhameed SA, Moussa SM, Khalifa ME. Privacy-preserving tabular data publishing: a comprehensive evaluation from web to cloud. Computers & Security. 2018;72:74–95.
13. Berdik D, Otoum S, Schmidt N, Porter D, Jararweh Y. A survey on blockchain for information systems management and security. Information Processing & Management. 2021;58(1):102397.
14. Deepa N, Pham QV, Nguyen DC, Bhattacharya S, Prabadevi B, Gadekallu TR, et al. A survey on blockchain for big data: approaches, opportunities, and future directions. Future Generation Computer Systems. 2022;131:209–226.
15. Wu PY, Cheng CW, Kaddi CD, Venugopalan J, Hoffman R, Wang MD. -Omic and electronic health record big data analytics for precision medicine. IEEE Transactions on Biomedical Engineering. 2016;64(2):263–273.
16. Wang Y, Kung L, Wang WYC, Cegielski CG. An integrated big data analytics-enabled transformation model: Application to health care. Information & Management. 2018;55(1):64–79.

</td></tr>
</table>

19. Nóbrega T, Pires CES, Nascimento DC. Blockchain-based privacy-preserving record linkage: enhancing data privacy in an untrusted environment. Information Systems. 2021;102:101826.

20. Ranbaduge T, Christen P, Schnell R; Springer. Secure and Accurate Two-Step Hash Encoding for Privacy-Preserving Record Linkage. Pacific-Asia Conference on Knowledge Discovery and Data Mining. 2020; p. 139–151.

21. Lazrig I, Ong TC, Ray I, Ray I, Jiang X, Vaidya J; IEEE. Privacy preserving probabilistic record linkage without trusted third party. 2018 16th annual conference on privacy, security and trust (PST). 2018; p. 1–10.

22. Stammler S, Kussel T, Schoppmann P, Stampe F, Tremper G, Katzenbeisser S, et al. Mainzelliste SecureEpiLinker (MainSEL): privacy-preserving record linkage using secure multi-party computation. Bioinformatics. 2022;38(6):1657–1668.

23. Karapiperis D, Gkoulalas-Divanis A, Verykios VS; IEEE. Distance-aware encoding of numerical values for privacy-preserving record linkage. 2017 IEEE 33rd International Conference on Data Engineering (ICDE). 2017; p. 135–138.

24. Schnell R, Borgs C; IEEE. Randomized response and balanced bloom filters for privacy preserving record linkage. 2016 IEEE 16th International Conference on Data Mining Workshops (ICDMW). 2016; p. 218–224.

25. Rao FY, Cao J, Bertino E, Kantarcioglu M. Hybrid private record linkage: Separating differentially private synopses from matching records. ACM Transactions on Privacy and Security (TOPS). 2019;22(3):1–36.

26. Zhang Y, Ng KS, Churchill T, Christen P. Scalable entity resolution using probabilistic signatures on parallel databases. Proceedings of the 27th ACM International Conference on Information and Knowledge Management. 2018; p. 2213–2221.

27. Vatsalan D, Yu J, Thorne B, Henecka W; Springer. P-signature-based blocking to improve the scalability of privacy-preserving record linkage. Data Privacy Management, Cryptocurrencies and Blockchain Technology: ESORICS 2020 International Workshops, DPM 2020 and CBT 2020, Guildford, UK, September 17–18, 2020, Revised Selected Papers 15. 2020; p. 35–51.

28. Yang Q, Shen Y, Vatsalan D, Zhang J, Kaafar MA, Hu W. P4mobi: A probabilistic privacy-preserving framework for publishing mobility datasets. IEEE Transactions on Vehicular Technology. 2020;69(7):6987–6999.

29. Ranbaduge T, Schnell R. Securing Bloom filters for privacy-preserving record linkage. Proceedings of the 29th ACM International Conference on Information & Knowledge Management. 2020; p. 2185–2188.

30. Smith D. Secure pseudonymisation for privacy-preserving probabilistic record linkage. Journal of Information Security and Applications. 2017;34:271–279.

31. Vidanage A, Ranbaduge T, Christen P, Schnell R; IEEE. Efficient pattern mining based cryptanalysis for privacy-preserving record linkage. 2019 IEEE 35th International Conference on Data Engineering (ICDE). 2019; p. 1698–1701.

32. Christen P, Schnell R, Ranbaduge T, Vidanage A. A critique and attack on "Blockchain-based privacy-preserving record linkage". Information Systems. 2021; p. 101930.

33. Vidanage A, Ranbaduge T, Christen P, Randall S. A privacy attack on multiple dynamic match-key based privacy-preserving record linkage. International Journal of Population Data Science. 2020;5(1).

34. Bernabe JB, Canovas JL, Hernandez-Ramos JL, Moreno RT, Skarmeta A. Privacy-preserving solutions for blockchain: Review and challenges. IEEE Access. 2019;7:164908–164940.

35. Frank E Harrell Jr TC. Titanic dataset; 2017. Available from: https://www.openml.org/d/40945.

36. Christen P. Preparation of a real temporal voter data set for record linkage and duplicate detection research. Te chnical Rep ort The Australian National University. 2014;.

37. Shannon C, Weaver W. The mathematical theory of communication, ed. R. Blahut, B. Hajek; 1998.

38. Karakasidis A, Verykios VS. Secure blocking+ secure matching= secure record linkage. Journal of Computing Science and Engineering. 2011;5(3):223–235.

39. Vatsalan D, Christen P, Verykios VS. A taxonomy of privacy-preserving record linkage techniques. Information Systems. 2013;38(6):946–969.

17. Jain P, Gyanchandani M, Khare N. Differential privacy: its technological prescriptive using big data. Journal of Big Data. 2018;5(1):1–24.

18. Vatsalan D, Sehili Z, Christen P, Rahm E. Privacy-preserving record linkage for big data: Current approaches and research challenges. Handbook of big data technologies. 2017; p. 851–895.

19. Christen P, Ranbaduge T, Vatsalan D, Schnell R. Precise and fast cryptanalysis for Bloom filter based privacy-preserving record linkage. IEEE Transactions on Knowledge and Data Engineering. 2018;31(11):2164–2177.

20. Heng Y, Armknecht F, Chen Y, Schnell R. On the effectiveness of graph matching attacks against privacy-preserving record linkage. PloS one. 2022;17(9):0267893.

21. Deng Y, Zeng Z, Jha K, Huang D. Problem-Based Cybersecurity Lab with Knowledge Graph as Guidance. Journal of Artificial Intelligence and Technology. 2021;.

22. Vidanage A, Christen P, Ranbaduge T, Schnell R. A graph matching attack on privacy-preserving record linkage. Proceedings of the 29th ACM International Conference on Information & Knowledge Management. 2020; p. 1485–1494.

23. Nóbrega T, Pires CES, Nascimento DC. Blockchain-based privacy-preserving record linkage: enhancing data privacy in an untrusted environment. Information Systems. 2021;102:101826.

24. Ranbaduge T, Christen P, Schnell R; Springer. Secure and Accurate Two-Step Hash Encoding for Privacy-Preserving Record Linkage. Pacific-Asia Conference on Knowledge Discovery and Data Mining. 2020; p. 139–151.

25. Sahu M, Padhy N, Gantayat SS, Sahu AK. Local binary pattern-based reversible data hiding. CAAI Transactions on Intelligence Technology. 2022;7(4):695–709.

26. Kumar P, Rahman M, Namasudra S, Moparthi NR. Enhancing security of medical images using deep learning, chaotic map, and hash table. Mobile Networks and Applications. 2023; p. 1–15.

27. Namasudra S, Devi D, Choudhary S, Patan R, Kallam S. Security, privacy, trust, and anonymity. Advances of DNA computing in cryptography. 2018; p. 138–150.

28. Lazrig I, Ong TC, Ray I, Ray I, Jiang X, Vaidya J; IEEE. Privacy preserving probabilistic record linkage without trusted third party. 2018 16th annual conference on privacy, security and trust (PST). 2018; p. 1–10.

29. Stammler S, Kussel T, Schoppmann P, Stampe F, Tremper G, Katzenbeisser S, et al. Mainzelliste SecureEpiLinker (MainSEL): privacy-preserving record linkage using secure multi-party computation. Bioinformatics. 2022;38(6):1657–1668.

30. Karapiperis D, Gkoulalas-Divanis A, Verykios VS; IEEE. Distance-aware encoding of numerical values for privacy-preserving record linkage. 2017 IEEE 33rd International Conference on Data Engineering (ICDE). 2017; p. 135–138.

31. Schnell R, Borgs C; IEEE. Randomized response and balanced bloom filters for privacy preserving record linkage. 2016 IEEE 16th International Conference on Data Mining Workshops (ICDMW). 2016; p. 218–224.

32. Rao FY, Cao J, Bertino E, Kantarcioglu M. Hybrid private record linkage: Separating differentially private synopses from matching records. ACM Transactions on Privacy and Security (TOPS). 2019;22(3):1–36.

33. Zhang Y, Ng KS, Churchill T, Christen P. Scalable entity resolution using probabilistic signatures on parallel databases. Proceedings of the 27th ACM International Conference on Information and Knowledge Management. 2018; p. 2213–2221.

34. Vatsalan D, Yu J, Thorne B, Henecka W; Springer. P-signature-based blocking to improve the scalability of privacy-preserving record linkage. Data Privacy Management, Cryptocurrencies and Blockchain Technology: ESORICS 2020 International Workshops, DPM 2020 and CBT 2020, Guildford, UK, September 17–18, 2020, Revised Selected Papers 15. 2020; p. 35–51.

35. Yang Q, Shen Y, Vatsalan D, Zhang J, Kaafar MA, Hu W. P4mobi: A probabilistic privacy-preserving framework for publishing mobility datasets. IEEE Transactions on Vehicular Technology. 2020;69(7):6987–6999.

36. Ranbaduge T, Schnell R. Securing Bloom filters for privacy-preserving record linkage. Proceedings of the 29th ACM International Conference on Information & Knowledge Management. 2020; p. 2185–2188.

37. Smith D. Secure pseudonymisation for privacy-preserving probabilistic record linkage. Journal of Information Security and Applications. 2017;34:271–279.

38. Gkoulalas-Divanis A, Vatsalan D, Karapiperis D, Kantarcioglu M. Modern privacy-preserving record linkage techniques: An overview. IEEE Transactions on Information Forensics and Security. 2021;16:4966–4987.

39. Al-Dulaimi A, Mumtaz S, Al-Rubaye S, Zhang S, Chih-Lin I. A framework of network connectivity management in multi-clouds infrastructure. IEEE Wireless Communications. 2019;26(3):104–110.

40. Vidanage A, Ranbaduge T, Christen P, Schnell R; IEEE. Efficient pattern mining based cryptanalysis for privacy-preserving record linkage. 2019 IEEE 35th International Conference on Data Engineering (ICDE). 2019; p. 1698–1701.

41. Hooshmand MK, Hosahalli D. Network anomaly detection using deep learning techniques. CAAI Transactions on Intelligence Technology. 2022;7(2):228–243.

42. Christen P, Schnell R, Ranbaduge T, Vidanage A. A critique and attack on "Blockchain-based privacy-preserving record linkage". Information Systems. 2022;108:101930.

43. Jain A, Srivastava N. Privacy-Preserving Record Linkage with Block-Chains. Cyber Security, Privacy and Networking. 2022; p. 61–70.

44. Vidanage A, Ranbaduge T, Christen P, Randall S. A privacy attack on multiple dynamic match-key based privacy-preserving record linkage. International Journal of Population Data Science. 2020;5(1):1345.

45. Bernabe JB, Canovas JL, Hernandez-Ramos JL, Moreno RT, Skarmeta A. Privacy-preserving solutions for blockchain: Review and challenges. IEEE Access. 2019;7:164908–164940.

46. Namasudra S, Roy P, Balusamy B, Vijayakumar P. Data accessing based on the popularity value for cloud computing. In: 2017 International Conference on Innovations in Information, Embedded and Communication Systems (ICIIECS). IEEE; 2017. p. 1–6.

47. Jr FEH, Cason T. Titanic dataset; 2017. Database: OpenML [Internet]. Available from: https://www.openml.org/d/40945.

48. Christen P. Preparation of a real temporal voter data set for record linkage and duplicate detection research. Te chnical Rep ort The Australian National University. 2014;.

49. Karakasidis A, Verykios VS. Secure blocking+ secure matching= secure record linkage. Journal of Computing Science and Engineering. 2011;5(3):223–235.

50. Vatsalan D, Christen P, Verykios VS. A taxonomy of privacy-preserving record linkage techniques. Information Systems. 2013;38(6):946–969.

[Comment 2-23]

Please give two paragraphs in the last section. One for concluding the entire chapter, and the second one for discussing future works. Also, complete it within 400 words.

(Response 2-23)

We appreciate the reviewers' valuable comments.

In response to Comment 2-23, we have revised the conclusion section of our manuscript to align with the suggestions provided. The section has been structured into two distinct paragraphs: the first one succinctly concludes the study, emphasizing the introduction and effectiveness of the zero-relationship encoding method designed for PPRL systems. We reiterated how this approach enhances privacy by preventing the direct correlation between the source

and encoded records, thereby mitigating the risk of re-identification.

In the second paragraph, we have addressed future works, outlining the areas where further research is needed. We discussed the optimization of the proposed method for handling larger datasets and more diverse environments, which would be beneficial in real-world applications. Additionally, we indicated our intention to explore further strategies to strengthen privacy protections within PPRL systems.

This revised structure ensures that the conclusion section comprehensively summarizes the study's findings while clearly delineating directions for future research. We believe that this modification not only adheres to the requested format but also provides a clear, coherent closure to our study, setting a defined pathway for subsequent research endeavors.

We hope that these revisions adequately address the reviewer's concerns and improve the overall quality of the manuscript.

The details of the revisions are as follows:

(Revision 2-23)

Before	After
[Section 5, p.12] This study proposes a zero-relationship encoding method designed to minimize the risk of record re-identification. The proposed method offers robust privacy preservation in a PPRL system while facilitating efficient record linkage. By eliminating the relationship between the source and encoded records, our approach complicates the task of reconstructing information from the encoded records. Therefore, even if an encoded record is leaked, it prevents re-identification and ensures privacy. The efficacy of the proposed method was substantiated by comparing its performance in re-identification attack simulations with those of existing encoding methods. Our method displayed superior resistance to re-identification attacks, drastically reducing the number of records that could be re-identified compared with conventional methods. Future studies should focus on optimizing the proposed method for larger datasets and diverse environments. Moreover, we plan to explore additional strategies to bolster privacy protections and construct a more secure PPRL system. In conclusion, the proposed zero-relationship encoding method	***[Section 5, p.12]*** In this study, we have introduced a zero-relationship encoding method aimed at minimizing the risk associated with record re-identification within PPRL systems. Our method significantly enhances privacy preservation by severing the link between source and encoded records, thus making the reconstruction of information from encoded data more challenging. The effectiveness of this approach was demonstrated through simulations of re-identification attacks, where our method exhibited superior resistance compared to existing encoding techniques. By considerably reducing the possibility of record re-identification, the proposed method ensures a higher degree of privacy protection in PPRL environments.<br>Future research should prioritize refining the proposed encoding technique for application in broader and more varied datasets. Additionally, further development is warranted to identify new measures that can fortify privacy safeguards even further, contributing to a more robust and secure PPRL framework. We are committed to continuing our exploration into methods that enhance the security of

reduces the potential for re-identification attacks in PPRL by eliminating the association between the source and encoded records, thus providing enhanced privacy preservation.	private information. By focusing on these areas, future studies will be able to build upon our findings and extend the capabilities of privacy-preserving techniques in record linkage systems, ensuring a balance between privacy protection and the efficiency of linkage processes.

[Comment 2-24]
Add section numbering

(Response 2-24)
We appreciate the reviewers' valuable comments.

In response to Comment 2-24 regarding the addition of section numbering, we have updated our manuscript to include clear and consistent section numbering throughout. This adjustment has been made to improve the document's structure and readability, ensuring that readers can more easily navigate and reference different parts of the paper. We hope that this change meets the requirements and enhances the overall presentation of our work.

The details of the revisions are as follows:

(Revision 2-24)

**Before**	**After**
[Section 1-6, p.1-12]<br>Introduction<br>Related Work<br>Methods<br>- Problem Formulation<br>- Zero-Relationship Encoding Method<br>Results: Evaluation and Discussion<br>- Simulation Setup<br>- Performance Evaluation<br>- Security Analysis<br>Conclusion<br>Acknowledgements	***[Section 1-6, p.1-12]***<br>1. Introduction<br>2. Related Work<br>3. Methods<br>3.1. Problem Formulation<br>3.2. Zero-Relationship Encoding Method<br>4. Results: Evaluation and Discussion<br>4.1. Simulation Setup<br>4.2. Performance Evaluation<br>4.3. Security Analysis<br>5. Conclusion<br>Acknowledgements

– Thank you so much –

[Response to Reviewer 3 Comments]

[Comment 3-1]
What were the simulation results for the Titanic dataset, and how many records, on average, were re-identified?

(Response 3-1)
We appreciate the reviewers' valuable comments.

The re-identification attack model and simulation results using the Titanic dataset, as presented by *Vidanage et al.,*[2] are as follows.

Encoding method	Match Algorithms		
	Symmetric	Hungarian	Shapley
Bloom filter [23]	87.24 % (1149)	86.48 % (1139)	87.31 % (1150)
Two-step hash [24]	94.98 % (1251)	94.98 % (1251)	94.98 % (1251)
Tabulation min-hash [37]	49.96 % (658)	51.17 % (674)	54.29 % (715)
Proposed scheme	1.36 % (18)	1.06 % (14)	1.36 % (18)

On average, the bloom filter re-identified 1,146 records, the two-step hash re-identified 1,251 records, and the tabulation min-hash re-identified 682.3 records. In contrast, our proposed method resulted in an average of only 16.6 re-identified records across all matching algorithms, the lowest among all. To aid in understanding the simulation outcomes, we have revised the visual materials to include graphs and numerical data on the results of the re-identification attacks.

The revisions are as follows:

(Revision 3-1)

Before	After

[2] Vidanage A, Christen P, Ranbaduge T, Schnell R. A graph matching attack on privacy-preserving record linkage. Proceedings of the 29th ACM International Conference on Information & Knowledge Management. 2020; p. 1485–1494

[Section 4.2, p.8] Figure 3. Re-identification rates in the Titanic dataset: Comparing encoding methods and matching algorithms.	***[Section 4.2, p.8]*** Figure 3. Re-identification rates in the Titanic dataset: Comparing encoding methods and matching algorithms.

[Section 4.2, p.8]

Figure 3. Re-identification rates in the Titanic dataset: Comparing encoding methods and matching algorithms.

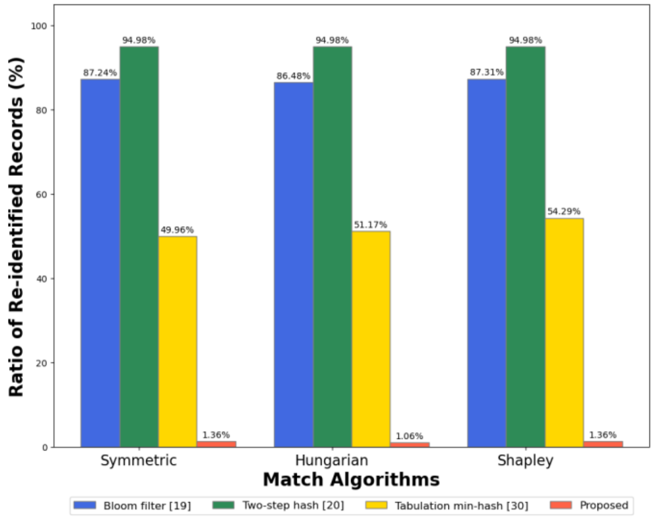

[Section 4.2, p.8]

Figure 3. Re-identification rates in the Titanic dataset: Comparing encoding methods and matching algorithms.

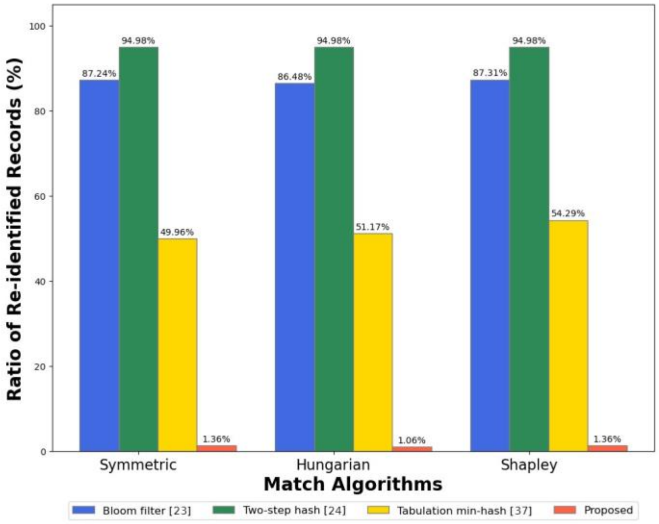

Encoding method	Match Algorithms		
	Symmetric	Hungarian	Shapley
Bloom filter [23]	87.24 % (1149)	86.48 % (1139)	87.31 % (1150)
Two-step hash [24]	94.98 % (1251)	94.98 % (1251)	94.98 % (1251)
Tabulation min-hash [37]	49.96 % (658)	51.17 % (674)	54.29 % (715)
Proposed scheme	1.36 % (18)	1.06 % (14)	1.36 % (18)

[Comment 3-2]

How does the security analysis support the claim that the zero-relationship encoding scheme preserves user privacy in the context of PPRL and graph-based re-identification attacks? Justify the same.

(Response 3-2)

We appreciate the reviewers' valuable comments.

In response to your feedback, we have extensively revised the security analysis section of our paper. We have elucidated the mechanisms through which our proposed encoding method fortifies privacy preservation against potential re-identification threats. Specifically, we have detailed how the zero-relationship encoding disrupts the direct correlation between source and encoded records, thus significantly complicating any attempts at unauthorized re-identification through graph-based analysis. By employing a combination of selective attribute utilization and complex encoding algorithms, our method effectively obfuscates the encoded data, rendering conventional re-identification techniques ineffective.

We believe these revisions provide a comprehensive justification of our claim and hope that you find the enhanced security analysis both convincing and in alignment with the paper's objectives.

The details of the revisions are as follows:

(Revision 3-2)

Before

[Section 4, p.9-12]

4.3. Security Analysis

In this section, we present a security analysis to validate the robustness and reliability of the proposed encoding method. The information-hiding ability of the proposed method and its defense performance against the graph-based re-identification attack model were analyzed. Furthermore, we determined whether the proposed encoding method, when applied to the PPRL system, could effectively address the standard threat model scenarios considered by the system.

4.3.1 Strong Privacy-Preservation

The privacy-preserving performance of the proposed PPRL system was analyzed using entropy. From the perspective of privacy protection, entropy serves as a measure of the degree of randomness or uncertainty in a dataset [37–39]. Entropy essentially signifies the complexity of the anonymization applied to the data, indicating the probability of the original data being inferred by a re-identification attack. A dataset with high entropy exhibits a high degree of randomness. This implies that each record appears with a similar probability or little overlap between records, making it challenging for an attacker to identify a specific entity precisely. Conversely, a dataset with a low entropy displays less randomness and is consequently more predictable, rendering it susceptible to re-identification attacks.

Using entropy, we can calculate the information gain (IG), which is a measure of how much more information an attacker obtains than the original information (information initially possessed or the state of having no information). In other words, we can numerically express using IG how devastating a specific attribute used in the encoding process is if it is leaked and used in a re-identification attack. Hence, we employed entropy and IG to quantitatively analyze the privacy-preserving performance of the proposed zero-relation encoding method. The entropy of a discrete random variable X, which represents the encoded dataset, is defined in Eq . A lower entropy of variable X implies higher certainty, thus a higher probability of predicting the value of X.

$$H(X) = -\sum_{x \in X} p(x) log_2 p(x)$$

Given the value of variable X, we can define the conditional entropy of the discrete random variable Y, which represents the selected attributes, as $H(Y|X)$. $H(Y|X)$ quantifies the uncertainty in predicting the value of Y given variable X (Eq **??**). IG measures the uncertainty of the information when inferring the source (Y) using information from the encoded record (X). The IG between the discrete random variables X and Y is defined in Eq . A lower IG value implies a greater difficulty in inferring the source from the encoded record, suggesting that the information contained

Table 2. Information Gain

Encoding method	Entropy	Match Algorithms		
		Symmetric	Hungarian	Shapley
Two-step hash [20]	3.77	0.283	0.347	0.376
Tabulation min-hash [30]	9.965	0.283	0.347	0.376
Proposed	17.77	0.283	0.347	0.376

in the variable (Y) does not significantly influence the re-identification of the encoded data.

$$IG(Y|X) = H(Y) - H(Y|X)$$

Table 2 presents the entropy and IG results calculated using the traditional encoding methods and the proposed zero-relation encoding method for PPRL. The bloom filter was excluded because it does not store any information regarding the order or frequency of the elements, making entropy and IG calculations impossible. The dataset encoded using the proposed method exhibited the highest entropy of 17.77, underscoring its capability to anonymize a dataset with randomness and effectively resist re-identification attacks. The IG values for the name, surname, and street address non-unique user attributes selected based on data frequency were calculated in the NCVR dataset. We observed that the more frequent an attribute is, the higher the IG value. This implies that if the street address with the highest IG value is used in the encoding process and subsequently leaked to an attacker, it would have the highest probability of being re-identified. Therefore, researchers or developers using an encoding method based on record attributes must consider the trade-off between the IG value of the attribute and the risk of unique information leakage.

4.3.2 Defense Against Graph-Based Re-Identification Attack

The graphical representations of encoded datasets are pivotal metrics for assessing the resistance of encoding methods to graph-based re-identification attacks. In the graphs, nodes correspond to a database's set of unique values, while edges indicate the similarities between these values. A high density of nodes means non-unique or similar values, making re-identification based solely on nodes more challenging. However, the presence of numerous edges can aid in identifying similarities between nodes, which can be exploited in re-identification attacks. Contrarily, dispersed nodes could also be problematic, as unique features may facilitate more straightforward identification. Ideally, an effective encoding method would produce a graph with fewer nodes and edges that are neither densely packed nor dispersed. The balanced distribution of graphs makes both feature extraction and re-identification more difficult. Using the Titanic and NCVR datasets, encoding was conducted using four techniques: bloom filter, two-step hash, table min-hash, and the proposed zero-relationship encoding method. Subsequently, the encoded datasets were transformed into graphical representations to assess their resistance to potential attacks visually. Fig 6 shows the characteristics of each encoding method when the encoded data are graphically represented.

- Bloom Filter and Two-Step Hash: For both the Titanic and NCVR datasets, these methods resulted in graphs with densely interconnected nodes, indicating that they possess inherent vulnerabilities to re-identification attacks.
- Tabulation Min-hash: The graph appears slightly less dense for the Titanic dataset than the graphs of the two previously discussed encoding methods. However, it still offers many extractable features, making it vulnerable to

After

[Section 4, p.10-13]

4.3. Security Analysis

This section addresses the robustness and reliability of our proposed zero-relationship encoding method in preserving user privacy against graph-based re-identification attacks within the context of PPRL systems. Our analysis focuses on the method's capacity to obfuscate information and its defensive efficacy against potential threats.

4.3.1. Strong Privacy-Preservation

The privacy-preserving efficacy of our proposed PPRL system is substantiated through an analysis utilizing entropy, a measure reflective of randomness or uncertainty within a dataset. Entropy signifies the complexity of the anonymization applied to the data, indicating the probability of the original data being inferred by a re-identification attack [49,50]. High entropy means a complex anonymization process, significantly reducing the likelihood of original data inference through re-identification attacks. This complexity confounds direct attacks and hinders advanced graph-based attacks by obfuscating the relationships within the data structure.

The entropy of a dataset is mathematically represented as:

$$H(X) = -\sum_{x \in X} p(x) \log_2 p(x),$$

where $H(X)$ denotes the entropy of a discrete random variable X, which encapsulates the encoded dataset, and $p(x)$ represents the probability of occurrence of an element x in X.

We extend this notion to calculate the information gain (IG), a metric quantifying the additional information an attacker gains post-attack. This is particularly relevant in assessing the vulnerability of specific attributes within the encoding process:

Table 3. Information Gain

Encoding method	Entropy	Match Algorithms		
		Symmetric	Hungarian	Shapley
Two-step hash [24]	3.77	0.283	0.347	0.376
Tabulation min-hash [37]	9.965	0.283	0.347	0.376
Proposed	17.77	0.283	0.347	0.376

$$IG(Y|X) = H(Y) - H(Y|X),$$

with $H(Y|X)$ being the conditional entropy of Y given X. A lower IG indicates a more resilient encoding scheme, implying that the attacker's knowledge does not significantly increase, even if certain attributes are compromised.

Our security analysis, as presented in Table 3, juxtaposes the entropy and IG values calculated for both traditional encoding methods and our proposed zero-relationship encoding scheme. Notably, the proposed method achieved a high entropy of 17.77, suggesting an enhanced level of data protection against re-identification.

To confront traditional methods' limitations, we highlight the superior entropy of our method, affirming its capacity to maintain data utility while providing strong privacy. This is contrasted with traditional approaches, which often manifest lower entropy, potentially compromising privacy.

In practical scenarios, such as health data exchange or customer data protection, the proposed method's high entropy and low IG values signal a significant advancement in mitigating the risks of sensitive data exposure. The method is especially pertinent for datasets with inherent attribute correlations, a common vulnerability in PPRL. Our careful selection and encoding of attributes reduce this risk, a crucial factor supported by our findings that higher frequency attributes yield greater IG values.

Conclusively, this rigorous quantitative analysis underscores the zero-relationship encoding method's robustness in theoretical metrics and practical resilience against graph-based re-identification attacks, cementing its role as a pivotal advancement in PPRL privacy measures.

4.3.2. Defense Against Graph-Based Re-Identification Attack

Graphical representations of encoded datasets serve as critical indicators of an encoding method's resilience to graph-based re-identification attacks. In these representations, nodes represent unique values from a database, and edges denote similarities among these values. A graph's resilience to re-identification attacks is inversely proportional to the density of its nodes and edges: denser graphs, with many interconnected nodes, make them vulnerable to attacks as they facilitate the extraction of similarities that can lead to re-identification. Conversely, dispersed nodes could pose challenges, as unique features in such a distribution may facilitate easier identification of individual records. Ideally, an effective encoding method would produce a graph with fewer nodes and edges that are neither densely packed nor dispersed.

In our analysis, we employed the Titanic and NCVR datasets to evaluate the resistance of various encoding techniques—including bloom filter, two-step hash, table min-hash, and our encoding method—against graph-based re-identification attacks. The encoded datasets were visually transformed into graphs, allowing for an assessment of each method's defensive capabilities. Fig 6 shows the characteristics of each encoding method when the encoded data are graphically represented.

- **Bloom Filter and Two-Step Hash:** These methods produced densely interconnected graphs for both the Titanic and NCVR datasets, revealing a

re-identification attacks. For the more complex NCVR dataset, the graph illustrates a densely packed node graph with many edges akin to those produced by the other two methods. This shows that the tabulation min-hash remains susceptible to graph-based re-identification attacks.

- Zero-Relationship Encoding: Our proposed method showcased exceptional resistance against re-identification. In the case of the Titanic dataset, the graph reveals nodes that are not densely packed but are uniformly spread with few edges. In contrast to the Titanic dataset, for the NCVR dataset, which inherently has a structured appearance owing to its multiple attributes, the graph still exhibited fewer edges and nodes clustered in multiple groups. This distribution makes feature extraction more challenging than the graphs derived from traditional encoding methods.

The graph representations show the robust defensive performance of the zero-relationship encoding method against graph-based re-identification attacks, particularly compared with other encoding methods.

fundamental susceptibility to re-identification attacks. The dense node connectivity in these graphs implies a higher risk of attribute similarity exploitation, which can lead to successful re-identification.

- **Tabulation Min-hash:** The graph for the Titanic dataset appeared slightly less dense compared to the previous methods, yet it still exhibited numerous extractable features that render it vulnerable to attacks. The NCVR dataset's graph, characterized by densely packed nodes and numerous edges, further confirmed the tabulation min-hash method's vulnerability to re-identification.
- **Zero-Relationship Encoding:** The zero-relationship encoding method demonstrated superior resistance to re-identification attacks. The Titanic dataset's graph, characterized by uniformly spread nodes with minimal edges, and the NCVR dataset's graph, with its nodes clustered into multiple groups and fewer edges, illustrate the method's effectiveness. The reduced node and edge density significantly complicates feature extraction and subsequent re-identification attempts, highlighting the zero-relationship encoding method's robustness.

In summary, the comparative analysis of graphical representations underscores the zero-relationship encoding method's enhanced defense against graph-based re-identification attacks. By minimizing node density and edge connectivity, our method substantially reduces the feasibility of exploiting attribute similarities, thereby preserving user privacy more effectively within PPRL frameworks.

4.3.3. Threat Model Scenario

PPRL systems facilitate the exchange of anonymized information among entities while safeguarding against unauthorized data breaches and re-identification threats. These systems, operating within a networked environment, are inherently exposed to a spectrum of security vulnerabilities, ranging from passive eavesdropping to active interference such as spoofing, sniffing, and snooping. The implications of such vulnerabilities extend to the unauthorized decryption and misuse of sensitive personal information, necessitating a robust defense mechanism inherent to the PPRL framework.

The design of the PPRL system is predicated on a semi-trust model among participating entities, who, while adhering to the established record linkage protocols, could potentially harbor honest-but-curious or outright malicious intents. The collaborative nature of the PPRL process involves the sharing of information necessary for record linkage, introducing a potential vector for the exploitation of anonymized data, thereby elevating the risk of re-identification without explicit consent from the data subjects.

Addressing the spectrum of threats, from honest-but-curious participants to external adversaries with malicious intent, the proposed zero-relationship encoding method employs a strategic limitation on the utilization of record attributes during the linkage process. This method leverages a selective subset of quasi-identifiers—attributes sufficiently generic yet vital for the linkage process such as name, sex, and age—thereby ensuring that the exposure of any single attribute does not compromise the integrity of the entire dataset. Moreover, the encoding process is further secured through the confidential agreement among participants on key parameters such as the hash function list and the attribute selection criteria, thereby obfuscating the encoding logic from potential adversaries.

The zero-relationship encoding approach fundamentally disrupts the direct correlation between the source and encoded records, thereby mitigating the risk associated with conventional encoding methodologies that preserve certain relational aspects susceptible to exploitation. By amalgamating multiple attributes to generate encoded values devoid of direct linkage to the original data, the method significantly impedes the adversaries' ability to apply cryptographic or pattern analysis techniques for data re-identification. This encoding paradigm, by obfuscating the relational mappings, effectively nullifies the utility of graph-based analysis tools in deciphering the encoded datasets, thereby fortifying the privacy assurances provided by the PPRL system.

In summary, the zero-relationship encoding method presents a comprehensive defense framework against the multifaceted threat landscape associated with PPRL systems. By ingeniously dissociating the encoded data from its source through a blend of attribute selection rigor and encoding complexity, the method not only thwarts attempts at unauthorized re-identification but also establishes a new benchmark for privacy preservation within the domain of PPRL.

[Comment 3-3]

In the North Carolina Voter Records (NCVR) dataset, what was the re-identification rate?

(Response 3-3)

We appreciate the reviewers' valuable comments.

The results of the re-identification attack simulation using the NCVR dataset are as follows.

Encoding method	Match Algorithms		
	Symmetric	Hungarian	Shapley
Bloom filter [23]	90.72 % (3993)	90.86 % (3999)	90.79 % (3996)
Two-step hash [24]	98.31 % (4327)	98.38 % (4330)	98.34 % (4328)
Tabulation min-hash [37]	40.58 % (1786)	40.17 % (1768)	40.94 % (1802)
Proposed scheme	0 % (0)	0 % (0)	0 % (0)

For the bloom filter, an average of 3,996 records were re-identified, the two-step hash re-identified an average of 4,328.3, and the tabulation min-hash re-identified 1,785.3 records. In the case of our proposed method, we observed zero re-identified records across all matching algorithms.

We reviewed the parameters to ascertain whether the dramatic results of our proposed method were due to errors or a failed attack process leading to erroneous outcomes. The feature extraction process from the graph of data encoded by our proposed method took an average of 1,718.59 seconds, up to 3.22 times longer than the extraction times for other encoding methods (BF: 542.84s, 2SH: 533.42s, TMH: 620.66s). The time taken for feature extraction indicates the difficulty of extracting features from encoded data, suggesting that our method effectively disrupts the relationships between information, thereby increasing the complexity of feature extraction and consequently making re-identification attacks more challenging.

Another metric we examined was the number of records incorrectly re-identified. In the case of the proposed methods, the re-identification attack on encoded 4,401 records. However, we confirmed that all records were matched with values entirely different from the originals, thus failing to identify the original records as intended. Our method involves selecting some attributes from the anonymized data and applying an XOR operation to produce values unrelated to the original, demonstrating an effective defense against attacks aiming to re-identify the original data from encoded features. To facilitate understanding of the simulation results, we have revised the visual materials to concurrently present both graphical and numerical data regarding the outcomes of the re-identification attacks.

The revisions are as follows:

(Revision 3-3)

**Before**	**After**

[Section 4.2, p.8]

Table 2. Re-identification rates in the NCVR dataset: Comparing encoding methods and matching algorithms.

Table 2. Re-identification rates in NCVR dataset: Comparing encoding methods and matching algorithms.

Encoding method	Match Algorithms		
	Symmetric	Hungarian	Shapley
Bloom filter [19]	87.24 %	86.48 %	87.31 %
Two-step hash [20]	94.98 %	94.98 %	94.98 %
Tabulation min-hash [30]	49.96 %	51.17 %	54.29 %
Proposed scheme	0 %	0 %	0 %

[Section 4.2, p.8]

Figure 4. Re-identification rates in the NCVR dataset: Comparing encoding methods and matching algorithms.

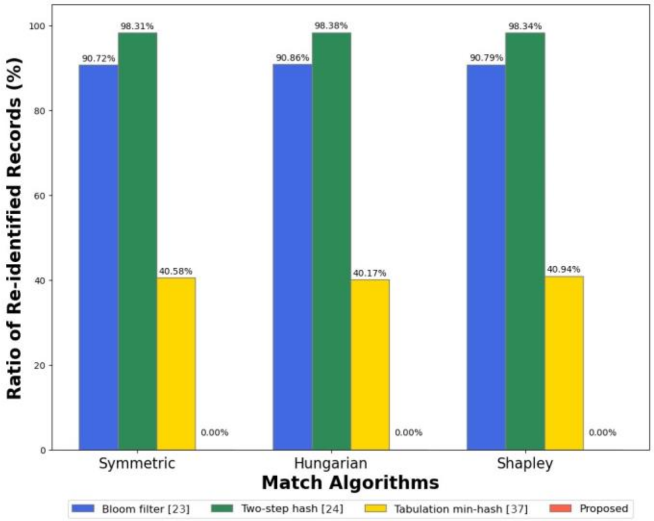

Encoding method	Match Algorithms		
	Symmetric	Hungarian	Shapley
Bloom filter [23]	90.72 % (3993)	90.86 % (3999)	90.79 % (3996)
Two-step hash [24]	98.31 % (4327)	98.38 % (4330)	98.34 % (4328)
Tabulation min-hash [37]	40.58 % (1786)	40.17 % (1768)	40.94 % (1802)
Proposed scheme	0 % (0)	0 % (0)	0 % (0)

[Comment 3-4]

How the results obtained indicate about the effectiveness of the zero-relationship encoding scheme?

(Response 3-4)

We appreciate the reviewers' valuable comments.

In response to your comments, we have further investigated the results of our re-identification attack simulations, affirming our encoding method's efficacy in minimizing the relational ties between original and encoded records, thereby deterring re-identification attacks. Our proposed approach, evidenced by simulations using the Titanic passenger dataset and the NCVR dataset, achieved notably low re-identification rates of 1.26% and 0%, respectively, compared to existing methodologies.

Our zero-relationship encoding scheme employs a method of XOR-folding selected quasi-identifier attributes to generate encoded records. The XOR operation combines various attributes through bit operations, generating arbitrary bit values unrelated to the original information. Consequently, the encoded records reference synthetic records that bear no resemblance to the original attributes, thus severing any direct relation to the original records.

Decoding to retrieve the original records necessitates knowledge of the specific types and parameters of attributes used in the encoding process. Should an attacker, lacking this crucial information, attempt re-identification, they would, at best, obtain values entirely disparate from the original records. Therefore, through its relationality-conscious encoding process, our method ensures that the outcomes of re-identification attacks are connected to incorrect val-

ues, thereby providing robust resistance against such attacks and privacy preservation.

– Thank you so much –
