# Supplementary material for: Secure privacy-preserving record linkage system from re-identification attack
Source: PLoS One. 2025 Jan 9;20(1):e0314486. doi: 10.1371/journal.pone.0314486 (PMC11717198; doi:10.1371/journal.pone.0314486)
Supplement: Attachment — Submitted filename: [PLOS ONE]Second revison_Reply Letter_Response to Reviewers.pdf [file pone.0314486.s004.pdf]

Reply Letter

Manuscript ID: PONE-D-23-43609

Title: Secure privacy-preserving record linkage system from re-identification attack

Authors: Sejong Lee, Yushin Kim, Yongseok Kwon, Sunghyun Cho*

Dear Associate Editor and Reviewers,

Most of all, we would like to thank the associate editor and the reviewers for spending their valuable time and effort reviewing our paper. We have carefully read all of the reviewers' comments and have revised our manuscript according to reviewers' insightful comments and suggestions. Please find below our detailed replies to each of the comments.

Once again, we appreciate your kind and careful suggestions.

Sincerely,

Sunghyun Cho, Prof./Ph.D.

Dept. of Computer Science and Engineering.
Hanyang University ERICA
55 Hanyangdaehak-ro, Sangrok-gu, Ansan, Gyeonggi-do, Korea.

Tel: +82-31-400-5670 / Fax: +82-31-436-8152
Email: chopro@hanyang.ac.kr

Authors' Response to Editor and Reviewers' Comments (Manuscript ID: PONE-D-23-43609)

[Response to Reviewer 1 Comments]

[Comment 1-1]
All figures have not good quality. You must improve the all figure quality. This is the basic need for any research paper.

(Response 1-1)
We appreciate the reviewers' valuable comments.

Regarding your concern about the figure quality, we would like to clarify that we have uploaded all figures in high resolution and in the format required by the journal to ensure the best possible quality. However, it appears that the PDF generation process on the submission site has caused some unintended degradation in the image quality.

We sincerely apologize for any inconvenience this may have caused. To address this issue, we will re-upload the figures and ensure that they maintain their original high resolution.

The uploaded images are as follows:

(Revision 1-1)

Figures

[Figure 1]

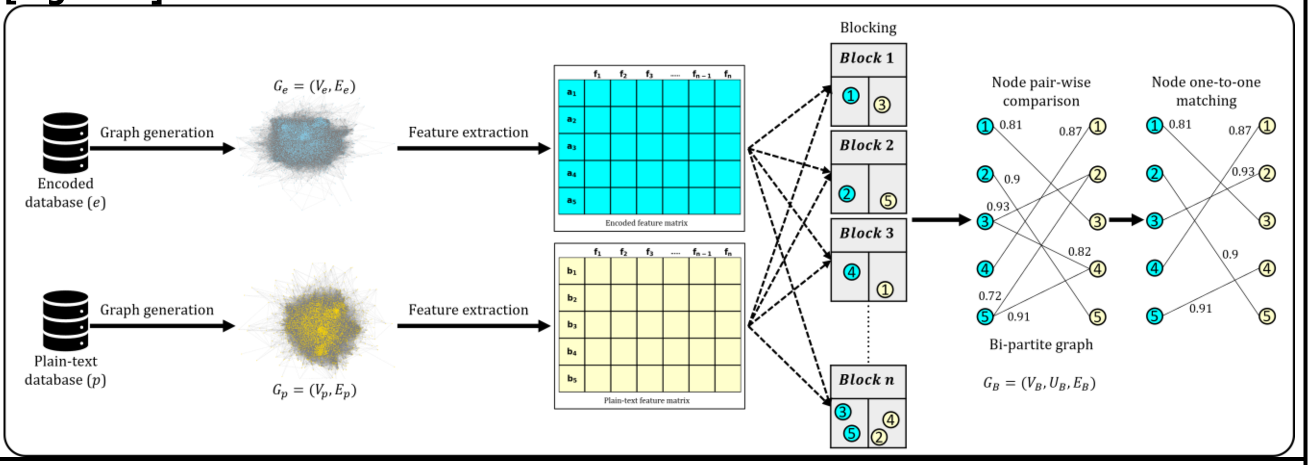

[Figure 2]

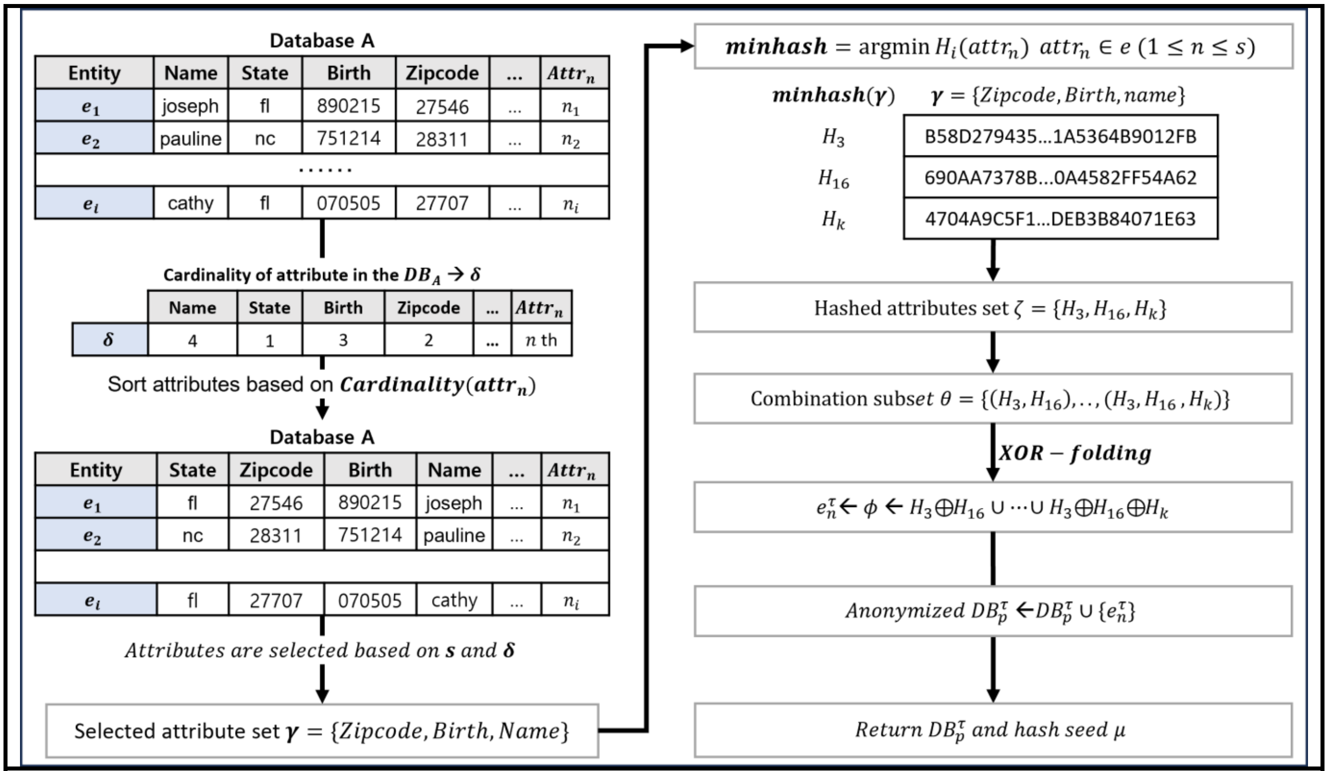

[Figure 3]

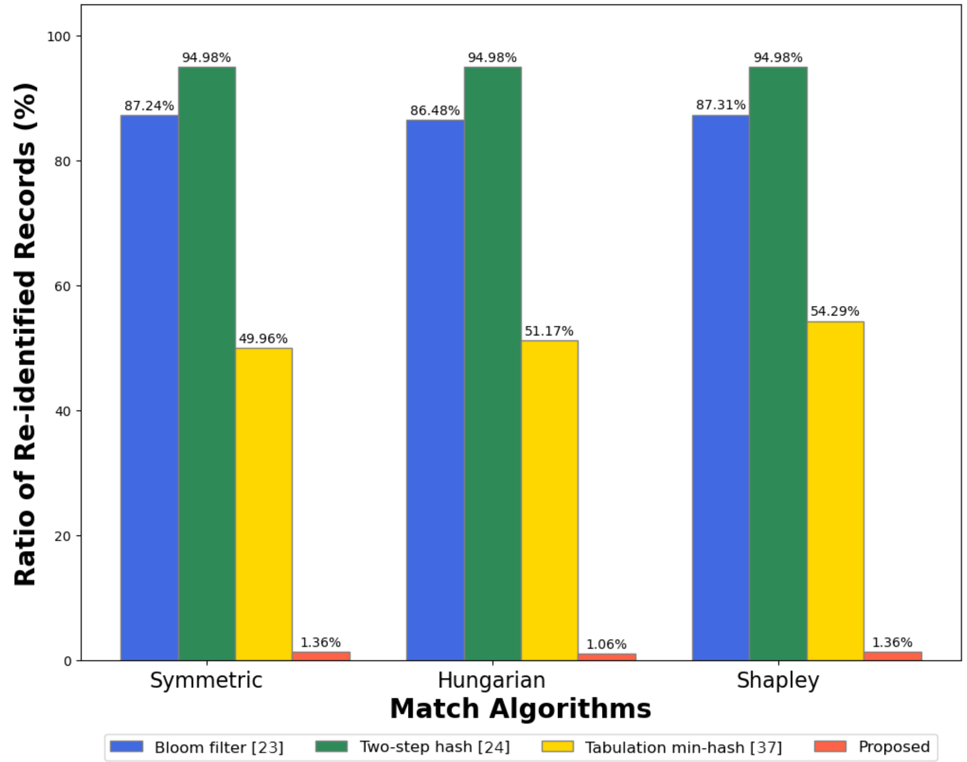

Encoding method	Match Algorithms		
	Symmetric	Hungarian	Shapley
Bloom filter [23]	87.24 % (1149)	86.48 % (1139)	87.31 % (1150)
Two-step hash [24]	94.98 % (1251)	94.98 % (1251)	94.98 % (1251)
Tabulation min-hash [37]	49.96 % (658)	51.17 % (674)	54.29 % (715)
Proposed scheme	1.36 % (18)	1.06 % (14)	1.36 % (18)

[Figure 4]

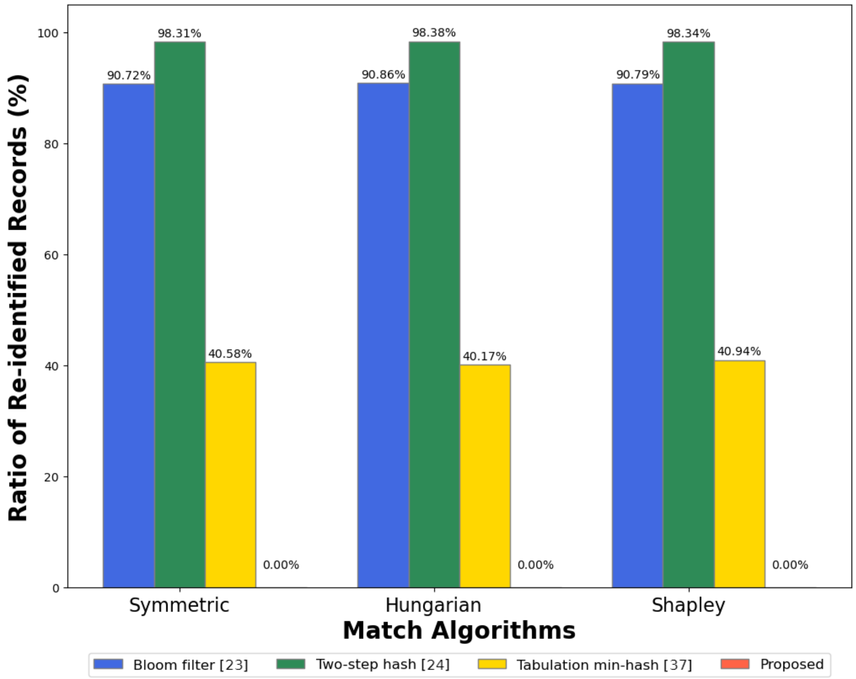

Encoding method	Match Algorithms		
	Symmetric	Hungarian	Shapley
Bloom filter [23]	90.72 % (3993)	90.86 % (3999)	90.79 % (3996)
Two-step hash [24]	98.31 % (4327)	98.38 % (4330)	98.34 % (4328)
Tabulation min-hash [37]	40.58 % (1786)	40.17 % (1768)	40.94 % (1802)
Proposed scheme	0 % (0)	0 % (0)	0 % (0)

[Figure 5]

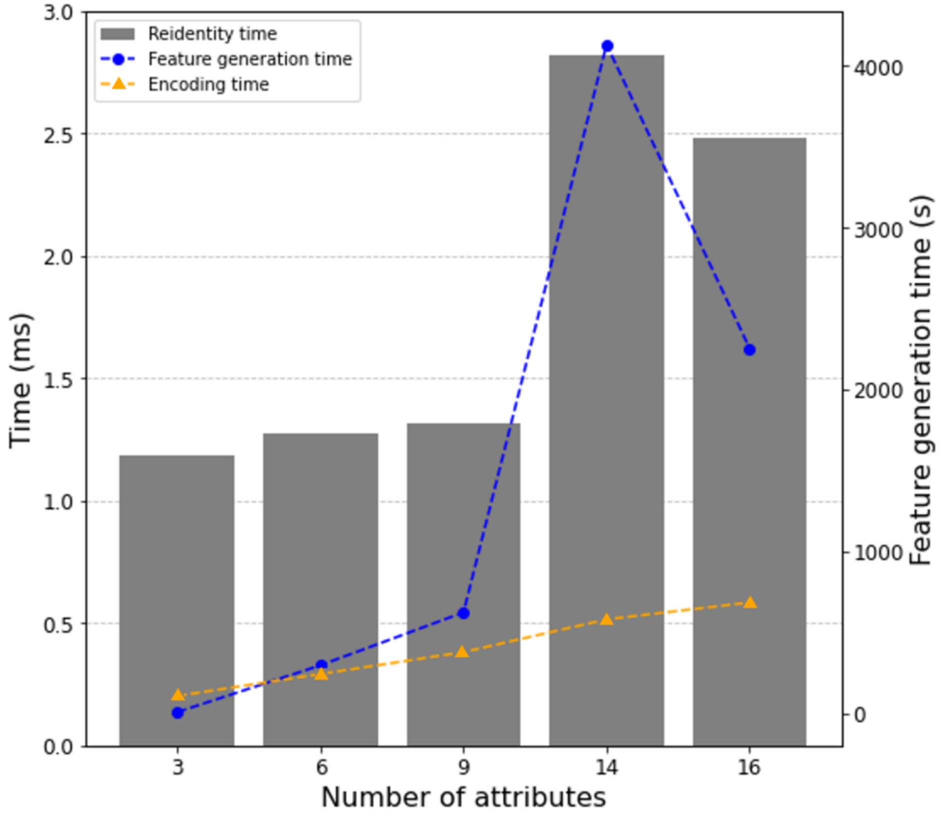

[Figure 6]

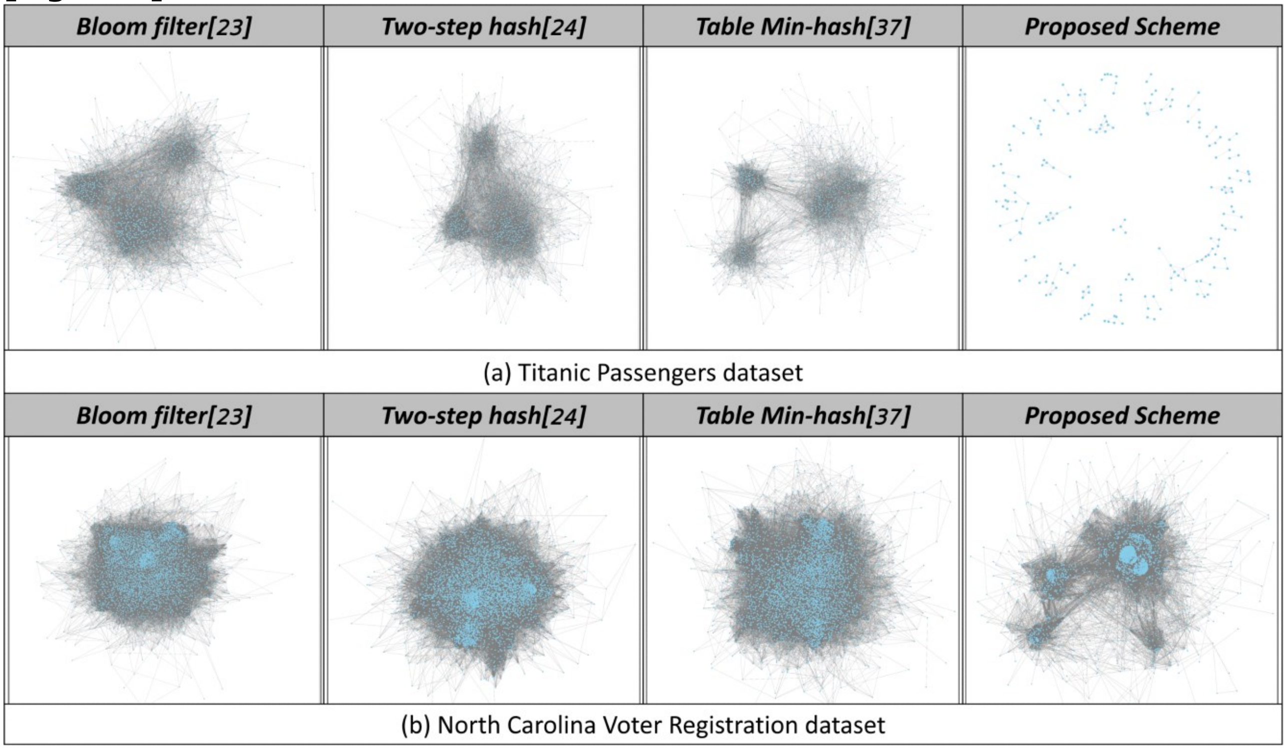

– Thank you so much –

[Response to Reviewer 4 Comments]

[Comment 4-1]
The comparison in Table 1 is too general. Please list the performance metrics considered in this article, and then analyze whether each method supports these metrics to make it more intuitive.

(Response 4-1)
We appreciate the reviewers' valuable comments.

Regarding your comment about the comparison in Table 1 being too general, we have revised the table to include specific performance metrics such as entropy and re-identification risk. We have analyzed these metrics to provide a more detailed and intuitive understanding of how each method supports these criteria. This update aims to offer a clearer comparison of the various approaches discussed in our paper.

Additionally, we have expanded the manuscript to include a discussion on the relationship between source data and encoded records during the encoding process. This discussion elaborates on the importance of entropy and re-identification risk as key factors in assessing the security of encoded data. We have highlighted that higher entropy increases data security by making it more unpredictable, while lower entropy and higher re-identification risk can pose significant privacy threats, especially when the relationship between source and encoded data is strong. By incorporating these elements, we aim to provide a more comprehensive understanding of the security challenges in privacy-preserving record linkage (PPRL) systems.

The details of the revisions are as follows:

(Revision 4-1)

Before	After
[Sec 2, p.4]<br>Existing studies have overlooked the relationship between the source and encoded records during the encoding process. The closer the relationship between the source and encoded records, the easier it is to decrypt them. An attacker can exploit this advantage to acquire the information required to infer the source data from the encoded data. Recent studies have demonstrated that attackers can identify a source record by launching a dictionary attack on the encoded record, provided they know the encoding parameters [20,22]. Therefore, developing a security method that considers the relationship between the source and encoded records is crucial during record encoding.	***[Sec 2, p.5]***<br>Existing studies have often overlooked the relationship between source data and encoded records during the encoding process. The closer this relationship, the easier it is for an attacker to decrypt the encoded data and infer the source data. This relationship is closely linked to the entropy and re-identification risk of the encoded data.<br><br>Entropy measures the randomness of the encoded data. Higher entropy indicates that the data is more unpredictable, making it more secure against attacks. Conversely, lower entropy means that the encoded data is more structured and predictable, increasing the likelihood of an attacker inferring the source data. This is particularly problematic when the relationship between the source data and encoded data is strong, as low entropy can make

Table 1. Related work to improve the security of PPRL

Year	Author	Description	Limitation
2017	Smith D.	• Proposed tabulation min-hash to remedy the BF encoding method vulnerabilities. Privacy preservation by avoiding simple mapping of plaintexts encoded into q-grams.<br>• Utilizes a bit array of length and lookup tables to encode records based on min-hash values of the q-gram set.	Maintaining essential bits for similarity in encoding.
2020	Ranbaduge et al.	• The windowing-based XORing method is proposed to mitigate vulnerabilities inherent in the BF encoding approach.<br>• Employs a sliding window to search for adjacent bit patterns within q-grams, subsequently combining these patterns using XOR to create new ones.<br>• The window size can be adjusted, allowing for pattern size alterations and optimizing the encoding process's efficiency and security.	Relationships between source and encoded records.
2020	Ranbaduge et al.	• Proposed a secure two-step hash encoding method. Hashes private record information into a bit matrix. Each column is hashed to generate an integer set representation of a sensitive input value.<br>• Real data simulations demonstrate improved record linkage quality in large databases.	Relationships between source and encoded records.
2021	Nóbrega et al.	• Assumed an unrealistic adversarial model and proposed an auditable blockchain-based PPRL.<br>• Highlights existing PPRL research limitations that rely on unrealistic adversarial models.<br>• To minimize information exchange during the record linkage process, the splitting bloom filters approach is proposed, which exchanges only parts of the encoded data, reducing security threats in the event of data breaches.	Re-identification risks arise if a user becomes malicious during BF exchange.
2022	Victor C. et al.	• Proposed an autoencoder-based method transforming BFs into numerical vectors, addressing vulnerabilities in BF encoding.<br>• Enhances security and privacy by preventing known BF attacks, offering a robust solution against re-identification risks.<br>• Data owners train autoencoder models for BF encoding/decoding, ensuring comparability via a mapping function.	Autoencoder sensitivity
2023	Siyu Y. et al.	• Proposed a PPRL using a Siamese neural network (SNN). SNN involves three main steps: combined bloom filter encoding module (CMF), blocking, and record matching using SNN. CMF combines a flat bloom filter (FBF) for strings and a transform bloom filter (TBF) for numerical attributes.<br>• Offers enhanced security via CMF encoding, precise record matching with SNN, adaptability to diverse data types, and robust performance in scenarios with data errors.	Scalability considerations and adaptation to data errors
2023	Frederik A. et al.	• Propose a BF with diffusion (BFD) PPRL scheme that approaches PPRL by extending conventional BFs with a linear diffusion layer.<br>• BFD enhances security against known attacks while preserving or improving linkage quality, potentially as a robust alternative to standard BF-based PPRL.	Complexity in balancing cost and fairness.
2024	Ours	• Proposes a zero-relationship encoding method to prevent re-identification attacks in PPRL and preserve privacy.<br>• Involves selecting quasi-identifiers based on their significance and applying XOR-folding to their hash values to generate encoded records, disrupting the re-identification process.<br>• Effectively protects privacy by preventing re-identification attacks and offers high efficiency due to low computational complexity in encoding.	Processing of records with a small number of attributes.

this inference easier.

Re-identification risk refers to the likelihood that a source record can be re-identified from the encoded data. A higher re-identification risk suggests that the encoded data is more likely to be linked back to the source data, posing a significant privacy threat. Recent studies have demonstrated that if an attacker knows the encoding parameters, they can identify a source record by launching a dictionary attack on the encoded record [20,22].

Therefore, it is crucial to develop a security method that maximizes entropy and minimizes re-identification risk during the encoding process, while also considering the relationship between source and encoded records. This approach ensures a balance between the randomness of the encoded data and the protection of the source data's privacy, making it harder for attackers to exploit any potential vulnerabilities.

Table 1. Related work to improve the security of PPRL

Year	Author	Description	Entropy	Identification risk	Limitation
2017	Smith D.	• Proposed tabulation min-hash to address vulnerabilities in BF encoding, enhancing privacy by preventing simple plaintext-to-q-gram mappings.<br>• Encodes records using min-hash values and a bit array to improve security.	Medium	Medium	Maintaining essential bits for similarity in encoding.
2020	Ranbaduge et al.	• Introduced a windowing-based XOR method to mitigate BF encoding vulnerabilities.<br>• Utilizes sliding windows to combine bit patterns within q-grams using XOR, with adjustable window sizes to optimize security and efficiency.	Medium	Low	Relationships between source and encoded records.
2020	Ranbaduge et al.	• Presented a two-step hash encoding method using a bit matrix for secure record linkage.<br>• Demonstrated improved linkage quality through simulations on large databases.	Medium	Low	Relationships between source and encoded records.
2021	Nóbrega et al.	• Proposed a blockchain-based PPRL approach to counter unrealistic adversarial models.<br>• Utilizes splitting bloom filters to reduce data exchange, minimizing security risks during record linkage.	Medium	Medium	Re-identification risks if a user becomes malicious during BF exchange.
2022	Victor C. et al.	• Proposed an autoencoder-based method to transform BFs into numerical vectors, enhancing security by preventing known BF attacks.<br>• Ensures data privacy and comparability through a mapping function for BF encoding and decoding.	High	Low	Autoencoder sensitivity.
2023	Siyu Y. et al.	• Proposed a PPRL method using a Siamese neural network (SNN) for secure and accurate record matching.<br>• Combines flat and transform bloom filters to handle diverse data types and improve performance in error-prone scenarios.	High	Very Low	Scalability considerations and adaptation to data errors.
2023	Frederik A. et al.	• Proposed a BF with diffusion (BFD) scheme, extending conventional BFs with a linear diffusion layer.<br>• Enhances security against known attacks while preserving or improving linkage quality.	High	Low	Complexity in balancing cost and fairness.
2024	Ours	• Proposes a zero-relationship encoding method to prevent re-identification attacks and preserve privacy.<br>• Applies XOR-folding to hash values for generating encoded records, enhancing security and efficiency.	Very High	Very Low	Processing of records with a small number of attributes.

[Comment 4-2]

The numbering of some formulas is missing and needs to be carefully modified.

(Response 4-2)

We appreciate the reviewers' valuable comments.

In response to your comments regarding the missing equation numbers, we have conducted a thorough review of all the equations in the manuscript. Initially, some equation numbers were removed based on a previous reviewer's suggestion to eliminate unnecessary numbering. However, considering your recent feedback, we have reinstated the numbering for those equations and ensured that all necessary equations are now correctly numbered throughout the manuscript.

These adjustments were made to provide a more comprehensive and precise presentation of the mathematical content, ensuring all equations are properly referenced for clarity and ease of understanding.

The details of the revisions are as follows:

(Revision 4-2)

<table>
<tr><th>Before</th><th>After</th></tr>
<tr>
<td>
[Sec 3.1, p.6]
$$\Xi(e_i^\tau, e_j^\tau) | \{e_i^\tau, e_j^\tau\} \in DB_i^\tau \times DB_j^\tau$$
The decision model compares and classifies all anonymized entities between the databases intended for linkage. The comparison process computes the similarity between entities using the Jaccard similarity measure and classifies them into matches (M) or mismatches (U) based on the computed similarity value.
$$\Xi(e_1^\tau, ...e_i^\tau) = \begin{cases} M \Leftrightarrow \forall(e_j^\tau, e_{j+1}^\tau) : (e_1^\tau, ...e_i^\tau), e_j^\tau = e_{j+1}^\tau \\ U \Leftrightarrow \exists(e_j^\tau, e_{j+1}^\tau) : (e_1^\tau, ...e_i^\tau), e_j^\tau \neq e_{j+1}^\tau \end{cases}$$
where(e_j^τ, e_{j+1}^τ) is anonymized entity set
[Sec 4.3.1, p.10]
$$H(X) = -\sum_{x \in X} p(x) \log_2 p(x)$$
where $H(X)$ denotes the entropy of a discrete random variable X, which
$$IG(Y|X) = H(Y) - H(Y|X)$$
</td>
<td>
[Sec 3.1, p.6]
$$\Xi(e_i^\tau, e_j^\tau) | \{e_i^\tau, e_j^\tau\} \in DB_i^\tau \times DB_j^\tau \quad (1)$$
The decision model compares and classifies all anonymized entities between the databases intended for linkage. The comparison process computes the similarity between entities using the Jaccard similarity measure and classifies them into matches (M) or mismatches (U) based on the computed similarity value.
$$\Xi(e_1^\tau, ...e_i^\tau) = \begin{cases} M \Leftrightarrow \forall(e_j^\tau, e_{j+1}^\tau) : (e_1^\tau, ...e_i^\tau), e_j^\tau = e_{j+1}^\tau \\ U \Leftrightarrow \exists(e_j^\tau, e_{j+1}^\tau) : (e_1^\tau, ...e_i^\tau), e_j^\tau \neq e_{j+1}^\tau \end{cases} \quad (2)$$
where(e_j^τ, e_{j+1}^τ) is anonymized entity set
[Sec 4.3.1, p.11]
$$H(X) = -\sum_{x \in X} p(x) \log_2 p(x) \quad (6)$$
where $H(X)$ denotes the entropy of a discrete random variable X, which encapsulates the encoded dataset, and $p(x)$ represents the probability of occurrence of an element x in X.
We extend this notion to calculate the information gain (IG), a metric quantifying the additional information an attacker gains post-attack. This is particularly relevant in assessing the vulnerability of specific attributes within the encoding process:
$$IG(Y|X) = H(Y) - H(Y|X) \quad (7)$$
</td>
</tr>
</table>

[Comment 4-3]

Some important literature discussions are missing, such as efficient privacy-preserving spatial range query over outsourced encrypted data, efficient privacy-preserving spatial data query in cloud computing, which suggests the author to supplement the discussion of these literatures. It is suggested that the author add some experiments to prove the advanced nature of the proposed scheme.

(Response 4-3)

We appreciate the reviewers' valuable comments.

Regarding your suggestion to include discussions on encrypted data queries in distributed environments and additional experiments, we agree that these are indeed important considerations. In fact, we have addressed these issues in a separate study that has been published in Sensors (https://www.mdpi.com/1424-8220/24/5/1582). This separate study focuses on privacy-preserving spatial range queries over outsourced encrypted data, specifically within

cloud computing environments, which aligns with the concerns you raised.

The current manuscript, however, is specifically focused on enhancing the security of PPRL systems by minimizing the re-identification risk through a zero-relationship encoding scheme. Given this targeted focus, we have not included a detailed discussion on encrypted data queries or the related experiments within this paper. To clarify this distinction, we have added a section in the manuscript explaining why these topics are not covered here and have referenced the related research in Sensors for readers interested in those aspects.

We believe that keeping the scope of each paper focused allows for a more thorough exploration of each specific issue. By addressing the re-identification prevention strategies in this manuscript and the encrypted data query handling in the separate paper, we aim to provide comprehensive insights into different facets of privacy-preserving technologies.

The details of the revisions are as follows:

(Revision 4-3)

**Before**	**After**
[Sec 2, p.2]<br>Among these, probabilistic methods have garnered attention because of their computational efficiency and capability to manage large-scale datasets [38]. However, security methods based on probabilistic approaches are vulnerable to privacy attacks, such as re-identification, inference, man-in-the-middle, and dictionary attacks [21]. Privacy leaks due to privacy attacks are a severe problem that undermines the purpose of PPRL [25–27]. Therefore, various methods have been proposed to overcome these limitations. We introduce some studies that are representative of improving the security performance of PPRL. Table 1 summarizes the contents of related works to strengthen PPRL security.	***[Sec 2, p.2]***<br>Among these, probabilistic methods have garnered attention because of their computational efficiency and capability to manage large-scale datasets [38]. However, security methods based on probabilistic approaches are vulnerable to privacy attacks, such as re-identification, inference, man-in-the-middle, and dictionary attacks [21] Privacy leaks due to privacy attacks are a severe problem that undermines the purpose of PPRL [25-27]. Therefore, various methods have been proposed to overcome these limitations.<br><br>While this study primarily focuses on enhancing the security of PPRL systems against re-identification attacks by minimizing the relationship between source and encoded records, it does not address the handling of encrypted data queries in distributed environments such as cloud computing. This aspect has been comprehensively explored in another study by the author, where an efficient privacy-preserving spatial range query for outsourced encrypted data was proposed. This study provides additional insights into managing and querying encrypted data in distributed environments, complementing the current research by focusing on different aspects of

	data security [39]. We introduce some studies that are representative of improving the security performance of PPRL. Table 1 summarizes the contents of related works to strengthen PPRL security.

– Thank you so much –
