# Supplementary material for: Secure privacy-preserving record linkage system from re-identification attack
Source: PLoS One. 2025 Jan 9;20(1):e0314486. doi: 10.1371/journal.pone.0314486 (PMC11717198; doi:10.1371/journal.pone.0314486)
Supplement: Attachment — Submitted filename: Secure privacy.docx [file pone.0314486.s001.docx]

Secure privacy-preserving record linkage (PPRL) system from re-identification attack

***Comments to authors:***

1. The title of this paper, you must modify. Short form is not allowed in title.
2. Improve your abstract section.
3. Which are you used software for simulation. Show the simulation results.
4. What is the novelty of your work? Include the comparison table with published work.
5. Atleast you mention Figures in result section like through flowchart etc.
6. How are you justify the equation 1-2.
7. Encoding and decoding is old techniques, why are you using this?
8. Elaborate the result section with using of proper results. This paper is like a review paper.
