# Supplementary material for: Secure privacy-preserving record linkage system from re-identification attack
Source: PLoS One. 2025 Jan 9;20(1):e0314486. doi: 10.1371/journal.pone.0314486 (PMC11717198; doi:10.1371/journal.pone.0314486)
Supplement: Attachment — Submitted filename: Review Comments_Reidentification attacks.docx [file pone.0314486.s002.docx]

This manuscript discusses a security vulnerability in PPRL technology, introduces a proposed solution in the form of a zero-relationship encoding scheme, and provides simulation results and security analysis to demonstrate the effectiveness of the proposed method in preserving user privacy and resisting graph-based re-identification attacks.

1. What were the simulation results for the Titanic dataset, and how many records, on average, were re-identified?

2. How does the security analysis support the claim that the zero-relationship encoding scheme preserves user privacy in the context of PPRL and graph-based re-identification attacks? Justify the same.

3. In the North Carolina Voter Records (NCVR) dataset, what was the re-identification rate?

4. How the results obtained indicate about the effectiveness of the zero-relationship encoding scheme?
